# Merging Bambus[6]uril and Biotin[6]uril into an Enantiomerically Pure Monofunctionalized Hybrid Macrocycle

**DOI:** 10.1021/acs.orglett.3c03715

**Published:** 2023-12-28

**Authors:** Arico Del Mauro, Jana Lapešová, Carola Rando, Vladimír Šindelář

**Affiliations:** † Department of Chemistry, Faculty of Science, Masaryk University, 625 00 Brno, Czech Republic; ‡ RECETOX, Faculty of Science, Masaryk University, 625 00 Brno, Czech Republic

## Abstract

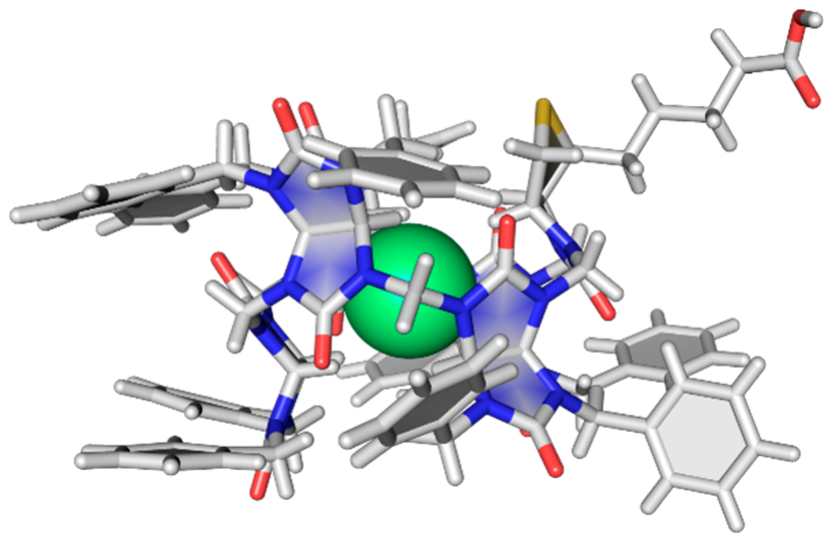

Bambus[6]urils and biotin[6]urils are macrocycles with an exceptional affinity for inorganic anions. Here, we investigated statistical condensation of 2,4-dibenzylglycoluril and d-biotin, monomers of the corresponding macrocycles, to prepare the enantiomerically pure macrocycle **1** containing a single d-biotin and five glycoluril units. Host–guest properties of **1** in chloroform solution and solid state were investigated. The macrocycle **1** bearing a single functional group was employed in the formation of [1]rotaxane utilizing reversible covalent bonds.

Bambus[6]urils (bambusurils in short) are macrocycles consisting of *six* glycoluril units, which are connected by *six* methylene bridges (Figure 1c).^1^ Bambusurils bind various inorganic anions inside their cavity due to 12 C–H···anion hydrogen bonds. Therefore, bambusurils are usually compared to hemicucurbiturils^2−6^ and biotin[6]uril (Figure 1a)^7,8^ macrocycles, which also contain ethyleneurea unit as a part of their building blocks and bind inorganic anions inside their cavity. Bambusurils are appreciated for their exceptional affinity and selectivity for many inorganic anions in organic solvents and water. For instance, water-soluble bambusuril derivatives bind small anions such as chloride and large iodide at millimolar and micromolar affinity, respectively, in water.^9,10^ Thus, bambusurils are investigated for their use in many areas including anion sensing,^11,12^ anion transport through lipophilic membranes,^13−15^ gold mining,^16^ hydrogel preparation,^17^ and others. The range of bambusuril applications can even be broadened as their supramolecular properties and solubility can be tuned by changing their substituents attached to nitrogen atoms at their portals^1^ or by substituting oxygen atoms of glycoluril building blocks by sulfur or nitrogen atoms.^18−21^

**Figure 1:**
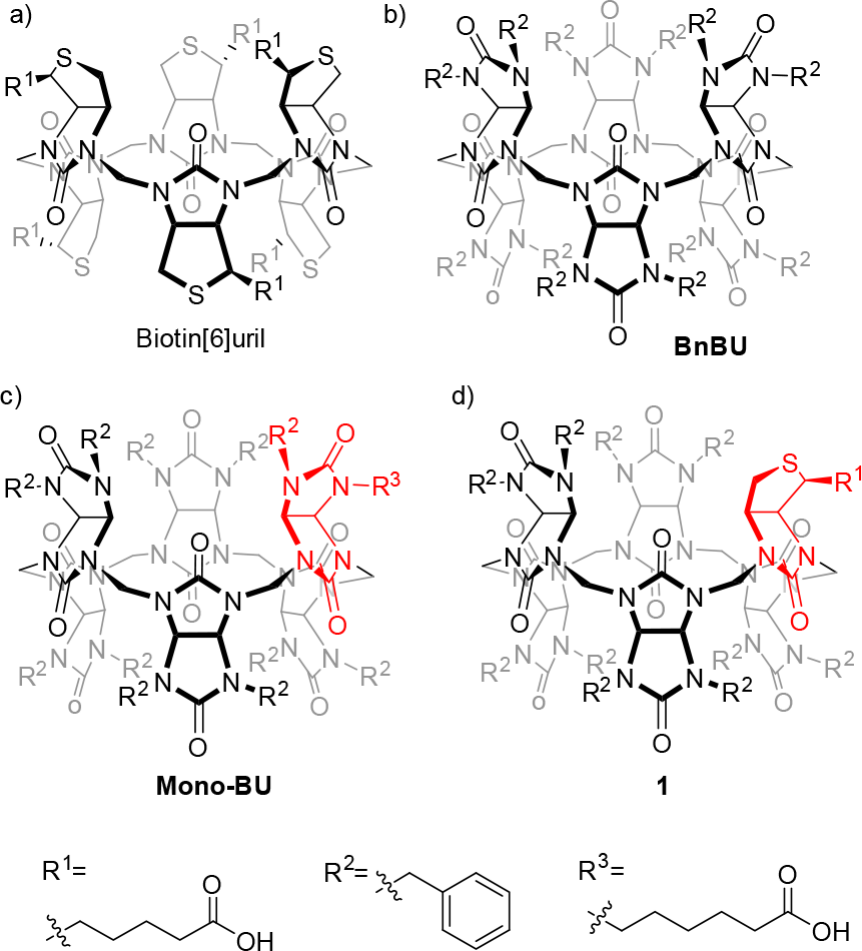
Chemical structures of (a) biotin[6]uril, (b) dodecabenzylbambus[6]urils (**BnBU**), (c) an example of a previously reported monofunctionalized bambus[6]uril (**Mono-BU**), and (d) hybrid macrocycle **1**.

Recently, we introduced monofunctionalized bambusurils (Figure 1c) and demonstrated their potential in the liquid–liquid extraction of anions, anion transport, and construction of mechanically interlocked molecules.^22−24^ The synthesis of monofunctionalized bambusurils is based on statistical condensation of formaldehyde with symmetrical glycoluril (such as 2,4-dibenzylglycoluril in **Mono-BU**, Figure 1c) in the presence of a small amount of unsymmetrical glycoluril (bearing a carboxyl group), which brings chirality into the system. To achieve enantiomerically pure monofunctionalized bambusurils, a single enantiomer of the unsymmetrical glycoluril must be used for the macrocyclization. However, the preparation of a single enantiomer of the glycoluril is rather difficult.^19^ Therefore, we envisioned that, in the macrocyclization reaction, the unsymmetrical glycoluril can be substituted for d-biotin, which is commercially available as a single enantiomer. Similarly to the 2,4-disubstituted glycolurils used for the bambusuril preparation, d-biotin contains two NH nitrogen functions, enabling its incorporation into the macrocycle. In this work, we report the preparation of monofunctionalized bambusuril **1**, in which one glycoluril is substituted for a d-biotin unit. The effect of the modification on the host–guest properties of **1** is investigated as well as its use in the preparation of [1]rotaxane.

The preparation of the macrocycle **1** was first tested following the reaction conditions optimized for previously published monofunctionalized bambusurils.^24^ A mixture of d-biotin and 2,4-dibenzylglycoluril was heated with paraformaldehyde and sulfuric acid in dioxane (Scheme 1), and the composition of the reaction mixture was followed by MALDI-TOF MS (Figure S17). The spectra showed the presence of **1** accompanied only by dodecabenzylbambus[6]uril (**BnBU**, Figure 1b), even when a relatively high d-biotin:glycoluril ratio of 1:5 was used for the reaction. This is in contrast with our previous work, in which such a high content of unsymmetrical glycoluril resulted in the formation of not only mono- but also di- and tri-substituted bambusurils.^22−24^ We also observed that in some macrocyclization attempts a small number of sulfur atoms of d-biotin were oxidized to sulfoxide during the reaction. Anion-free monofunctionalized bambusuril **1** was obtained in 35% yield after the crude mixture was boiled in aqueous solution of NH_3_ and purified by flash chromatography.

**Scheme 1:**
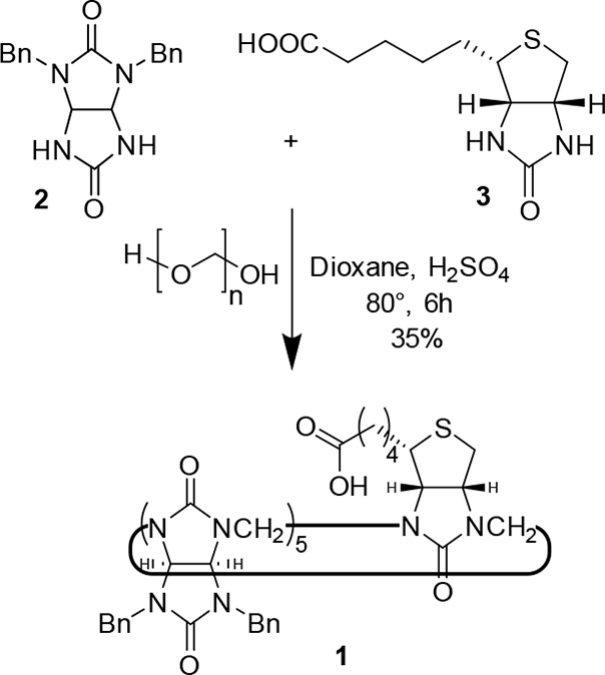
Synthesis of Macrocycle **1**

Diffusion of diethyl ether vapor into a solution of **1** and tetrabutylammonium chloride in chloroform resulted in colorless monocrystals suitable for X-ray diffraction analysis. The determined crystal structure confirmed that the macrocycle consists of one d-biotin unit and five glycoluril units (Figure 2). It also showed that the methine hydrogen atoms of the d-biotin unit direct to the cavity center while participating in the anion binding. As typical for bambusuril complexes, **1** binds the chloride anion inside its cavity, where it is stabilized by 12 C–H···Cl^–^ hydrogen bonding interactions with the average distance of 2.955 Å. One portal of **1** is occupied by a molecule of chloroform. The stabilization of the chloroform molecule is due to C–Cl···Cl^–^ halogen bond interaction (3.289 Å) with the bound chloride anion and C–H···S hydrogen bond interaction (2.206 Å) with the sulfur atom of the d-biotin unit (Figure 2b). The second portal of **1** engulfs the carboxyl group of the second molecule of **1**, while the group interacts with the encapsulated chloride anion through COO–H···Cl^–^ hydrogen bonding. Thus, the crystal structure is represented by two molecules of **1** differing in their orientation that self-assemble into helixlike supramolecular polymeric chains.

**Figure 2:**
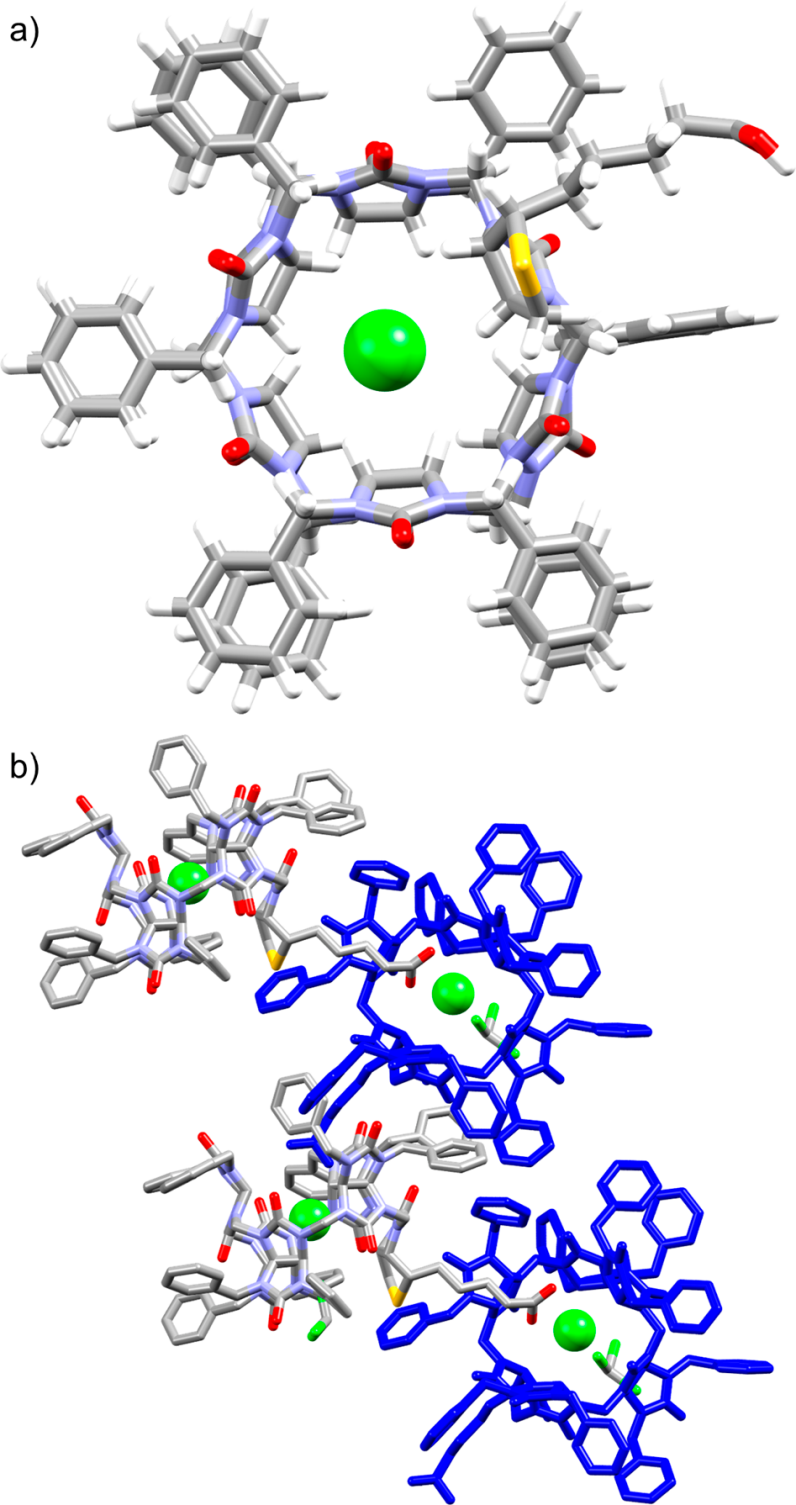
Crystal structure of the Cl^–^⊂**1** complex. (a) Top view and (b) arrangement of the complex into supramolecular polymeric chains.

The supramolecular properties of **1** in solution were studied by ^1^H NMR spectroscopy and isothermal titration calorimetry (ITC). First, we investigated a possible self-assembly of **1** similar to that observed in the solid state. ^1^H NMR spectra of **1** in chloroform and acetonitrile with the concentration increasing up to 30 mM did not show any broadening or concentration-induced chemical shifts. Similar results were obtained for chloride complexes in chloroform, where the absence of self-assembly was further confirmed by diffusion-ordered spectroscopy experiments (Figures S14–S16). Second, we wanted to evaluate the influence of the d-biotin unit on the host–guest properties of **1**. We previously reported association constants of **BnBU** complexes with various anions in chloroform.^25^**BnBU** differs from **1** just by a single dibenzylglycoluril unit, which is substituted by a d-biotin unit in **1**. Thus, we studied complexes of **1** and model anions MeSO_3_^–^, Cl^–^, Br^–^, and I^–^ in chloroform by ITC and compared them to the corresponding complexes of **BnBU** (Table 1). The results showed a 1:1 binding stoichiometry for all of the investigated systems. Macrocycle **1** forms the weakest complex with MeSO_3_^–^, and the stability of its complexes increases further for halides in the row Cl^–^, Br^–^, and I^–^. Differences in binding affinities can be explained by the anion solvation energy, which is significantly higher for MeSO_3_^–^ and Cl^–^ compared to Br^–^ and I^–^.^25^

**Table 1:** Association Constants (*K*_a_) and Thermodynamic Parameters of 1:1 Complexes between Selected Anions and Macrocycles **1** Determined by ITC (CHCl_3_, 298.15 K)^a^

	Macrocycle **1**			**BnBU**
Anion	Δ*H* (kJ mol^–1^)	*T*Δ*S* (kJ mol^–1^)	*K*_a_ (M^–1^)	*K*_a_ (M^–1^)
MeSO_3_^–^	–35.9	–6.5	1.4 × 10^5^	7.3 × 10^5^
Cl^–^	–54.8	–11.3	4.4 × 10^7^	1.3 × 10^7^
Br^–^	–63.9	–11.7	1.4 × 10^9^	6.7 × 10^8^
I^–^	–68.6	–12.4	7.3 × 10^9^	1.6 × 10^10^

a *K*_a_ of previously reported^25^ anion complexes with **BnBU** are given for comparison.

All investigated host–guest events were driven by enthalpy compensated by the entropic term. Similar host–guest characteristics were previously observed for the complexes of **BnBU**. Absolute values of association constants of the **1** and **BnBU** complexes are relatively similar. The largest difference was found for the complexes of MeSO_3_^–^, in which the anion is bound by **BnBU** about 5 times stronger compared to **1**. These results showed that incorporation of d-biotin into the bambusuril structure does not significantly influence its binding ability toward anions.

Recently, we used monofunctionalized bambusurils bearing a carboxyl function on their alkyl substituent for the construction of [1]rotaxanes utilizing a bis(acyloxy)iodate(I) reversible covalent bond.^24^ Macrocycle **1** features a similar substituent as the monofunctionalized bambusurils. Therefore, we decided to demonstrate the potential of **1** by its transformation into an interlocked molecule. We studied the formation of [1]rotaxane *in situ* using ^1^H NMR. The stoichiometric amount of **1** and bis(acyloxy)iodate(I) was dissolved in CD_3_CN (Figure 3), which resulted in significant changes in the ^1^H NMR spectrum compared to both reagents. The most characteristic signal of the rotaxane formation was an upfield shift (Δσ = 0.6 ppm) of methyl protons H(B) of bis(acyloxy)iodate(I). Furthermore, methine signals of the macrocycle became sharper, more distinguishable, and significantly shifted from their original position. Careful analysis of **1** employing ROESY measurement (Figure S13) revealed that only six methine protons show cross peaks with the acetoxy methyl group protons. Although the complexity of the NMR spectra precluded assignment of the methine protons, we assume that the methyl group of the axle interacts with six methine protons positioned in the lower part of the macrocycle. Similar cross peaks in ROESY spectra were observed for the previously published [1]rotaxane.^24^ Furthermore, an upfield shift of proton H(A) of 0.8 ppm was observed after the addition of bis(acyloxy)iodate(I) (Figure 3) as a consequence of folding of the aliphatic substituent into the cavity of the macrocycle. All the characteristics discussed above are in agreement with the formation of [1]rotaxane.

**Figure 3:**
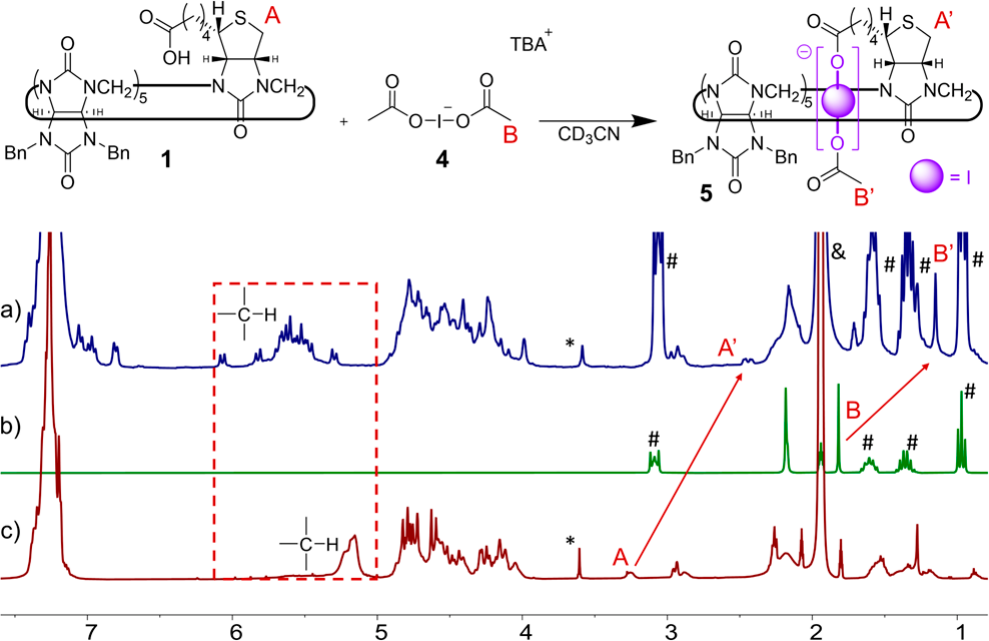
^1^H NMR (500 MHz, CD_3_CN, 298.15 K) spectra of (a) the [1]rotaxane **5** formed quantitatively *in situ*, (b) bis(acyloxy)iodate(I) **4**, and (c) macrocycle **1**. *Dioxane, ^#^tetrabutylammonium, ^&^acetonitrile.

In conclusion, we synthesized macrocycle **1** as the first hybrid of bambus[6]urils and biotin[6]uril macrocycles. Selective arrangement of the building blocks into a macrocyclic structure resulted in the macrocycle containing one d-biotin and five glycoluril units. Furthermore, d-biotin introduced one carboxyl function, enabling selective functionalization of the macrocycle. This was demonstrated by the formation of [1]rotaxane. Binding affinity and selectivity of **1** toward inorganic anions were similar to those of **BnBU**. The iodide⊂**1** complex was the most stable one with an association constant of 7.3 × 10^9^ M^–1^ in chloroform. In the solid state, molecules of **1** self-assemble into a helical supramolecular polymer through inclusion of carboxyl groups of one molecule into the portal of a neighboring molecule of the macrocycle. Our study also showed that the enantiomerically pure monofunctionalized bambusurils **1** can be used for the preparation of [1]rotaxane.

## Data Availability

The data underlying this study are available in the published article and its Supporting Information.

## Supporting Information Available

General methods, synthesis of compounds, NMR spectra, MALDI TOF spectra, isothermal titration calorimetry (ITC) data, and crystallography data (PDF)
