# Supplementary material for: Merging Bambus[6]uril and Biotin[6]uril into an Enantiomerically Pure Monofunctionalized Hybrid Macrocycle
Source: Org Lett. 2023 Dec 28;26(1):106–9. doi: 10.1021/acs.orglett.3c03715 (PMC10789090; doi:10.1021/acs.orglett.3c03715)
Supplement: Supplementary file 1 — ol3c03715_si_001.pdf [file ol3c03715_si_001.pdf]

Supporting Information

Merging Bambus[6]uril and Biotin[6]uril into Enantiomerically Pure Monofunctionalized Hybrid Macrocycle

Arico Del Mauro, Jana Lapešová, Carola Rando, Vladimír Šindelář*

Department of Chemistry, Faculty of Science, Masaryk University, 625 00 Brno, Czech Republic and RECETOX, Faculty of Science, Masaryk University, 625 00 Brno, Czech Republic

*sindelar@chemi.muni.cz

Table of Contents

1. **General Methods** **S2**
2. **Synthesis of compounds** **S3**
3. **NMR Spectra** **S6**
4. **MALDI TOF spectra** **S16**
5. **Isothermal Titration Calorimetry (ITC)** **S18**
6. **Crystallography** **S21**
7. **References** **S26**

1. General Methods

All reagents and deuterated solvents were purchased from commercial suppliers and used without further purification. HPLC and deuterated solvents were further dried over 4 Å molecular sieves. Reaction mixtures were heated on DrySyn heating blocks, and the reaction temperatures stated refer to the settings of the magnetic stirrer.

NMR spectra were recorded on a Bruker Avance III 300 MHz, and Bruker Avance III 500 MHz spectrometer. Chemical shifts (in ppm) are referenced to residual solvent peaks of deuterated solvent. Standard abbreviations for multiplicity are used as follows: s = singlet, d = doublet, t = triplet, q = quartet, dd = doublet of doublets, m = multiplet, and br = broad. Structural assignments were made with additional information from COSY, HSQC, HMBC, ROESY, and DOSY experiments.

ITC analysis was recorded on MicroCal VP-ITC from Malvern.

HRMS analysis was recorded on Agilent 6224 Accurate-Mass TOF LC-MS. Samples were ionized by electrospray ionization (ESI) or atmospheric pressure chemical ionization (APCI). Matrix assisted laser desorption ionization with detection of time of flight (MALDI-TOF) mass spectra were measured on the MALDI-TOF MS UltrafleXtreme (Bruker Daltonics). Samples were ionized by Nd-YAG laser (355 nm) from 2,5-dihydroxybenzoic acid (DHB) matrix. Melting points were measured on Stuart SMP40 melting point apparatus.

Diffraction data were collected on a Rigaku MicroMax-007 HF rotating anode CCD diffractometer and the structures were solved by direct methods and refined by full matrix least-squares methods using SHELXT and SHELXL.

Chromatographic separations were carried out on a CombiFlash® NextGen 300+ (Teledyne ISCO) instrument.

2. Synthesis of compounds

Synthesis of 2,4-dibenzylglycoluril

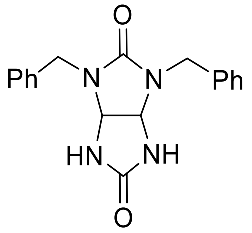

The synthesis was performed according to the literature procedure.[1] A mixture of 1,3-dibenzylurea (50 g, 0.21 mol) and 4,5-dihydroxyimidazol-2-one (36.86 g, 0.31 mol) was suspended in a solution of methanol (300 mL) and 35% HCl (3 mL). The suspension was heated to reflux, and complete dissolution of the starting material occurred within 1 hour. The reaction was concluded in 5.5 hours. After cooling to RT, the product was precipitated by slowly adding water. The resulting suspension was left in a beaker overnight. Additional water was then added, and the precipitate was collected by filtration and washed with water. The crude product was suspended in water, heated to reflux, and stirred vigorously for 1 hour. The suspension was collected by filtration and washed with water. The crystalline product was isolated with a yield of 81%.

^{1}H NMR (300 MHz, DMSO-d_6, 298.15 K): δ=7.61 (s, 2H,), 7.37-7.25 (m, 10H,), 5.07 (s, 2H,), 4.34 (dd, J=15.6, 175.2 Hz, 4H,)

All data correspond to those in the literature.[1]

Synthesis of **4**

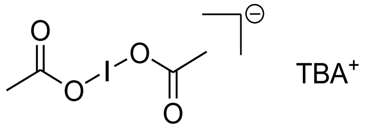

According to the literature procedure[2], (diacetoxyiodo)benzene (100 mg, 0.31 mmol) and tetrabutylammonium iodide (TBAI, 137.61 mg, 0.37 mmol) were weighted in separate vials, evacuated, and flushed with argon 5 times. TBAI was dissolved in dry $CDCl_3$ (1 ml) and transferred with a double-tipped needle to the vial containing (diacetoxyiodo)benzene. The solution was stirred at room temperature under argon atmosphere overnight. The reaction was quenched by adding dry diethyl ether (1 mL). The solution was transferred to dry diethyl ether (12 mL), and a slightly yellow precipitate was formed. The precipitate was isolated using 5 cycles of centrifugation (8000 rpm, 5 min), washing with dry diethyl ether, and dried under high vacuum. The spectra conform to the literature.[3]

Yield: 145 mg (96 %)

^{1}H NMR (300 MHz CD_3CN-d_3) δ 3.13 – 3.02 (m, 8H), 1.82 (s, 6H), 1.59 (m, J = 7.9 Hz, 8H), 1.43 – 1.26 (m, 9H), 0.97 (t, J = 7.3 Hz, 12H).

Synthesis of **1**

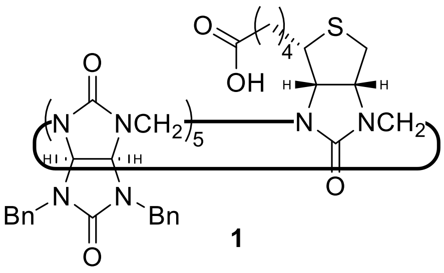

1

D-Biotin (0.347 g, 1.39 mmol), 2,4-dibenzylglycoluril (2.25 g, 6.97 mmol, 5.00 eq.) and paraformaldehyde (0.315 g, 10.5 mmol, 7.50 eq.) were dissolved in dry dioxane (11 mL; ca. 5 mL/g of monomers) containing conc. H_2SO_4 (0.330 mL). The reaction mixture was heated to 80 °C, and stirred at 80 °C for 4 h. Then it was cooled to RT and the precipitate was collected by filtration, washed with dioxane (4 mL) and dried *in vacuo*. The crude mixture (2.09 g) was transferred to a 250 mL flask and suspended in MeOH (20 mL), milli-Q water (20 mL) and aqueous solution of NH_3 (25 %, 15 mL). The mixture was refluxed for 2 h and then stirred for 24 h at room temperature. The precipitate was collected by filtration, washed with milli-Q water (15 mL) and MeOH (15 mL), and dried *in vacuo*. The precipitate containing anion-free dodekabenzylbambus[6]uril and **1** was separated by automated flash chromatography using 2 % methanol in dichloromethane as a mobile phase. After drying, a white solid was obtained.

Yield: 938 mg (35%)

M_p > 140°C (decomp.)

^{1}H NMR (500 MHz, DMSO-d_6) δ 12.00 (s, 1H), 7.24–7.06 (m, 50H), 5.37 – 5.04 (m, 10H), 4.91 – 3.95 (m, 34H), 3.48 (m, 1H), 3.03 (s, 1H), 2.88 (s, 1H), 2.21 (t, *J* = 7.2 Hz, 2H), 1.62 – 1.40 (m, 4H), 1.29 (s, 2H).

$^{13}C\{^1H\}$ NMR (126 MHz, DMSO-d_6) δ 174.3, 128.5, 128.4, 127.1, 126.9, 126.7, 47.2, 33.6, 24.2.

MALDI-TOF (+)MS *m/z*: $[M+Na]^+$ Calcd for $C_{106}H_{106}N_{22}O_{13}SNa$ 1949.7923; Found 1949.7904.

$[\alpha]^{23}_{589}$= –8.12 ° (c = 0.51 g/ 100 ml, $CHCl_3$).

In situ Preparation of **5**

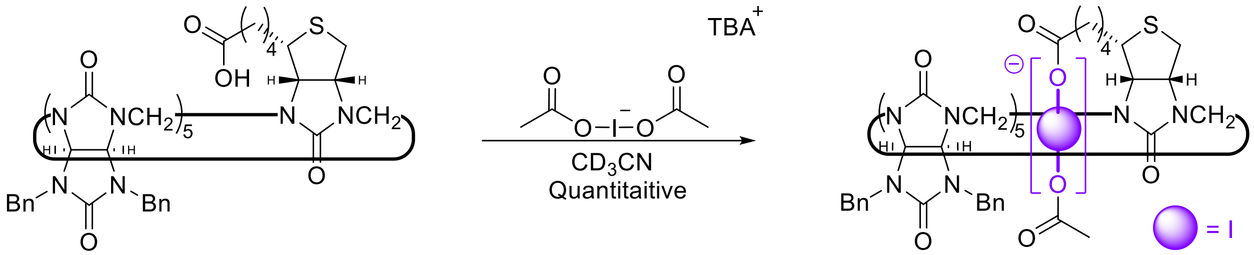

[1]Rotaxane **5** was synthesized *in situ* in NMR tube, requiring only a few minutes of reaction time. The reaction was initiated by mixing a solution of **4** (0.5 mL, 5 mM, 1 equiv.) in CD_3CN-d_3 with the solution of **5** (5 mg, 2.5 μmol) in CD_3CN-d_3.

^{1}H NMR (500 MHz, CD_3CN-d_3, 298.15 K) δ 7.32 – 6.77 (m, 50H), 6.06 (d, *J* = 8.5 Hz, 1H), 5.82 (d, *J* = 8.6 Hz, 1H), 5.70 – 5.46 (m, 7H), 5.31 (d, *J* = 8.7 Hz, 7H), 4.87 – 3.96 (m, 34H), 3.08 – 3.05 (m, 8H), 2.46 (dd, *J* = 13.4, 4.8 Hz, 1H), 2.26 – 2.23 (m, 2H), 1.62 – 1.56(m, 10H), 1.35 (h, *J* = 7.4 Hz, 8H), 1.16 (s, 3H), 0.96 (t, *J* = 7.4 Hz, 12H).

^{13}C{^{1}H} NMR (126 MHz, CD_3CN-d_3, 298.15 K) δ 172.5, 162.0, 160.5, 159.8, 140.4, 129.4, 129.4, 129.4, 129.3, 127.9, 127.8, 127.5, 127.3, 127.0, 70.6, 70.4, 59.4, 59.4, 59.3, 48.2, 34.3, 34.1, 30.3, 29.5, 28.8, 24.3, 20.7, 20.3, 20.3, 20.3, 13.8.

ESI (-) MS *m/z*: $[M]^-$ Calcd for $C_{108}H_{108}IN_{22}O_{15}S$ 2112.6; Found 2112.7.

3. NMR Spectra

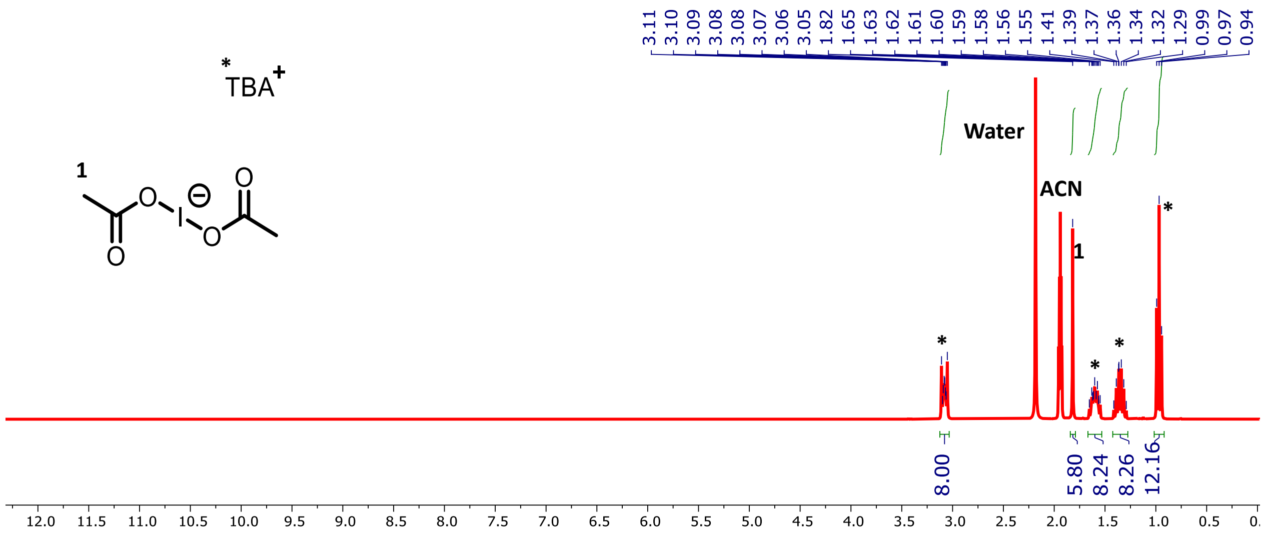

Figure S1. ^{1}H NMR spectrum (300 MHz, CD_3CN, 298.15 K) of **4**.

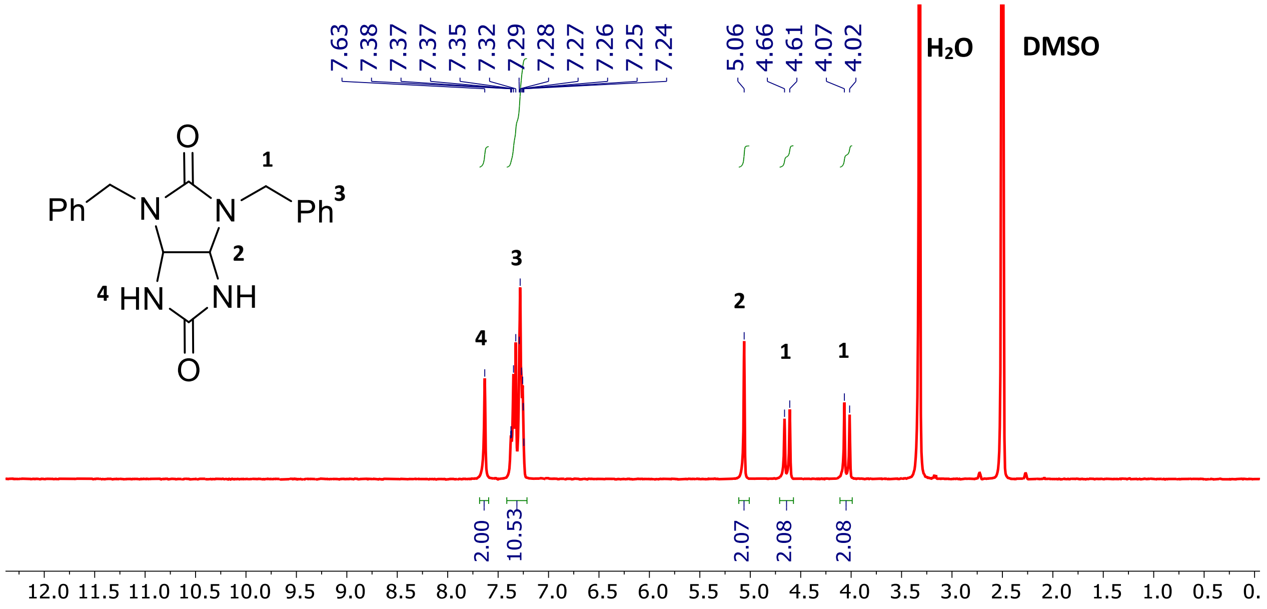

Figure S2. ^{1}H NMR spectrum (300 MHz, DMSO, 298.15 K) of 2,4-dibenzylglycoluril.

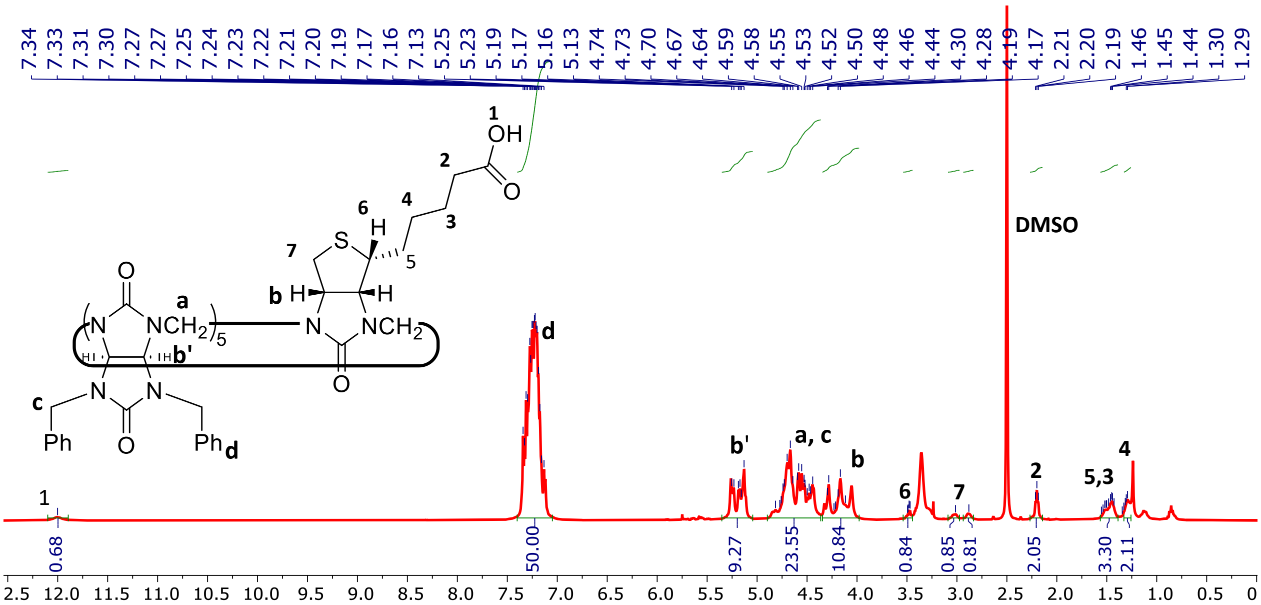

Figure S3. ^{1}H NMR spectrum (500 MHz, DMSO-d_6, 298.15 K) of **1**.

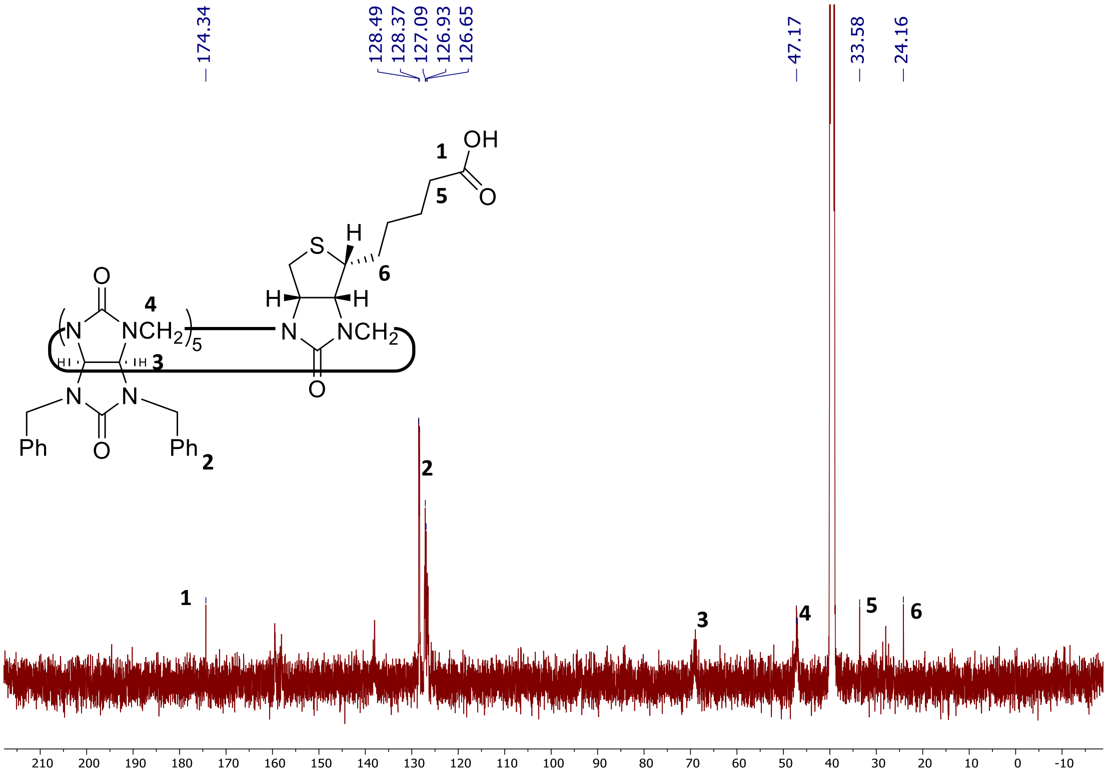

Figure S4. ^{13}C NMR spectrum (126 MHz, DMSO-d_6, 298.15 K) of **1**.

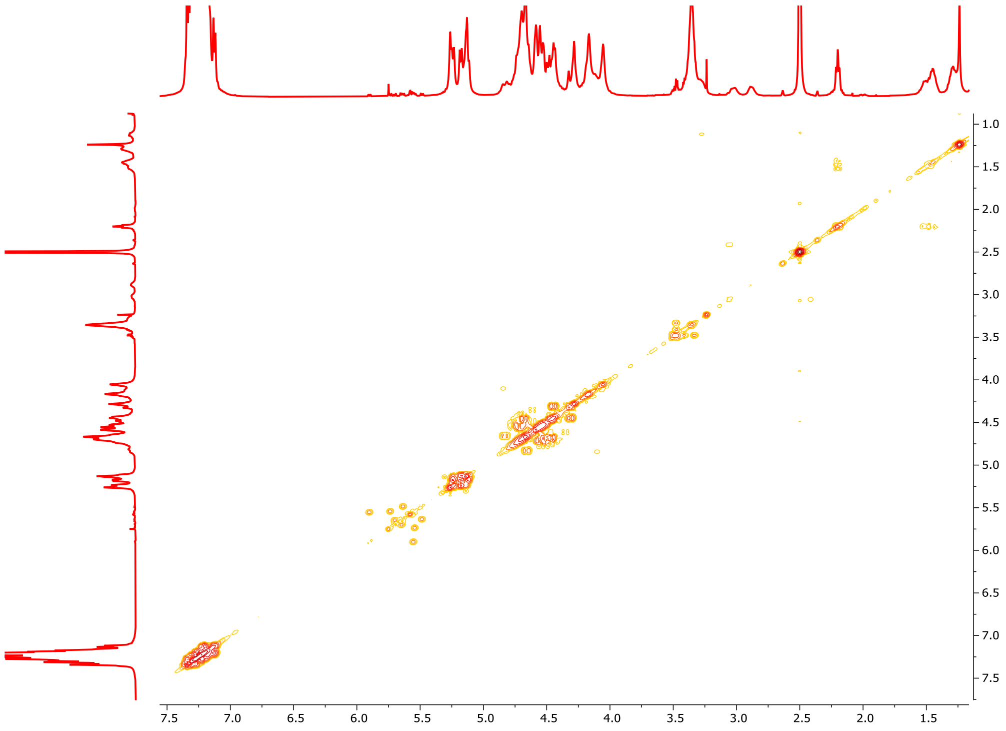

Figure S5. COSY NMR spectrum (500 MHz, DMSO-d_6, 298.15 K) of **1**.

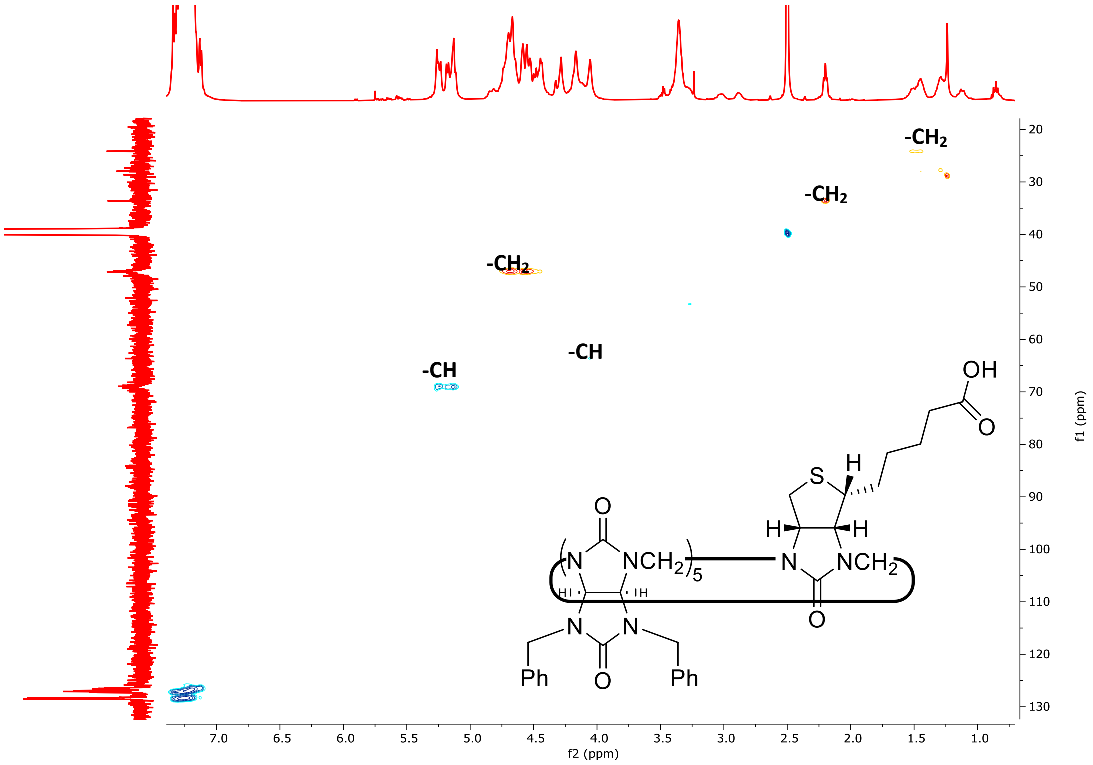

Figure S6. HSQC NMR spectrum (500 MHz, DMSO-d_6, 298.15 K) of **1**.

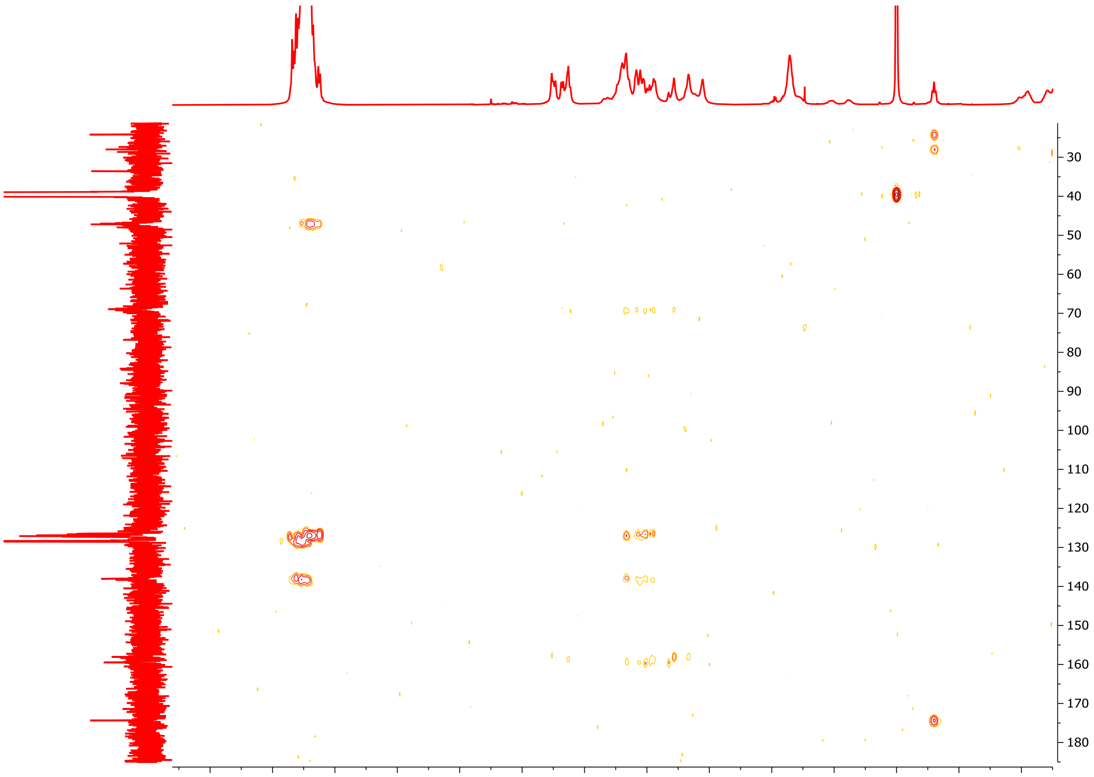

Figure S7. HMBC NMR spectrum (500 MHz, DMSO-d_6, 298.15 K) of **1**.

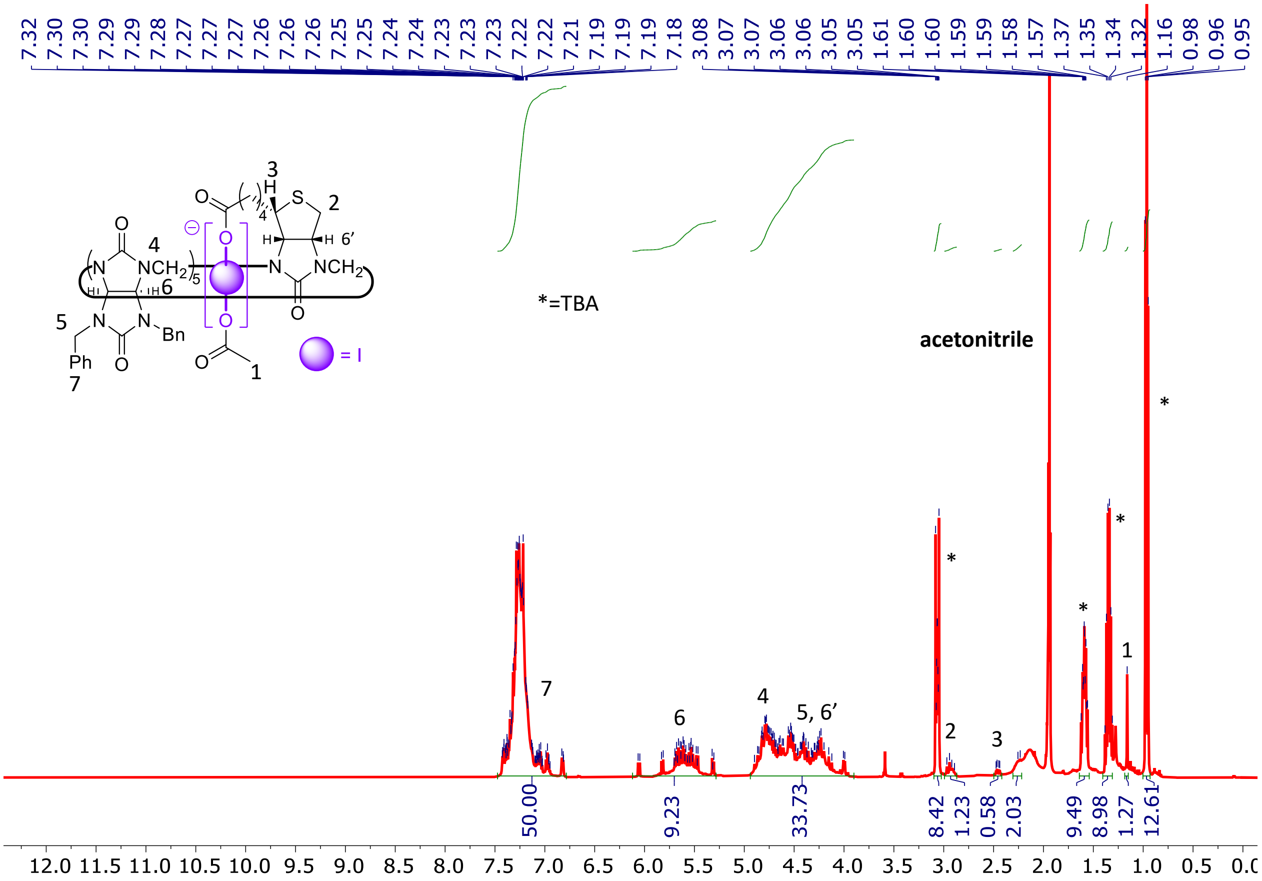

Figure S8. ^{1}H NMR spectrum (500 MHz, CD_3CN-d_3, 298.15 K) of **5** formed quantitatively *in situ*.

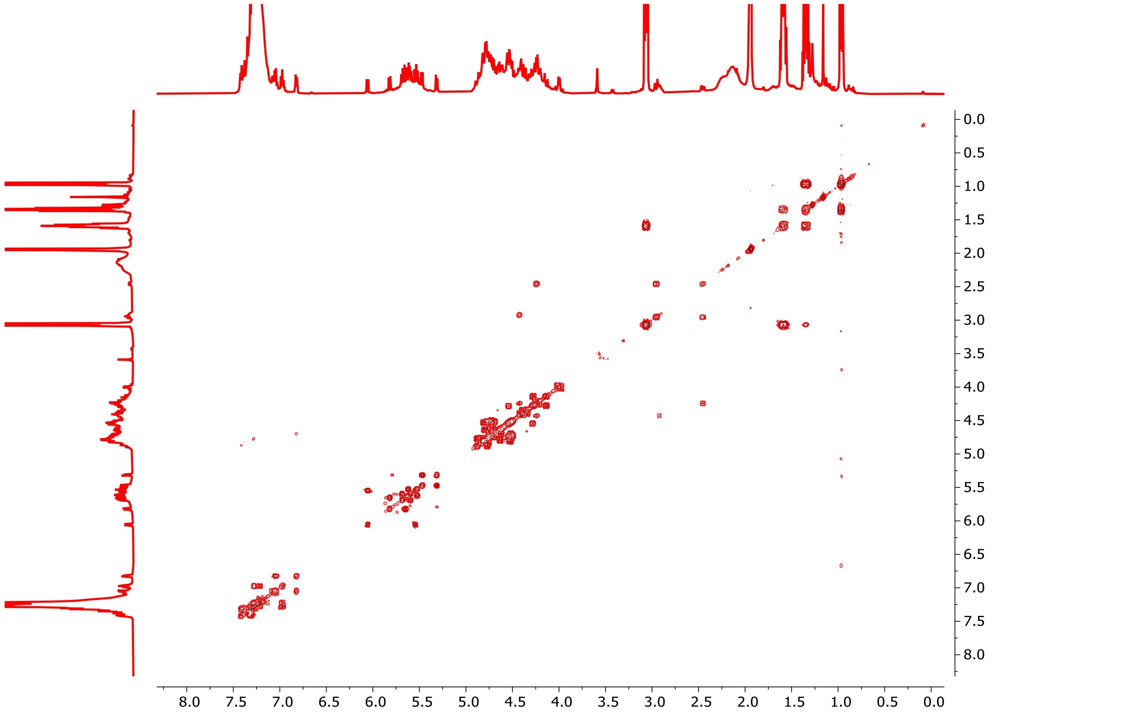

Figure S9. COSY NMR spectrum (500 MHz, CD_3CN-d_3, 298.15 K) of **5**.

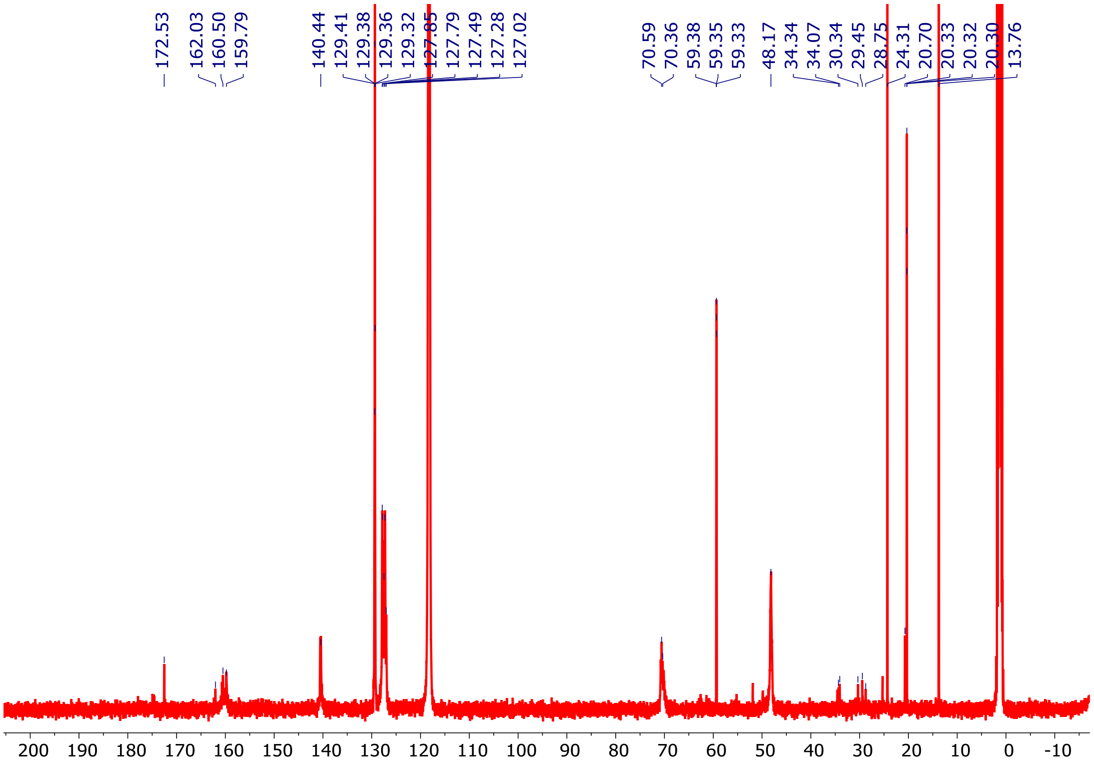

Figure S10. ^{13}C NMR spectrum (126 MHz, CD_3CN-d_3, 298.15 K) of **5**.

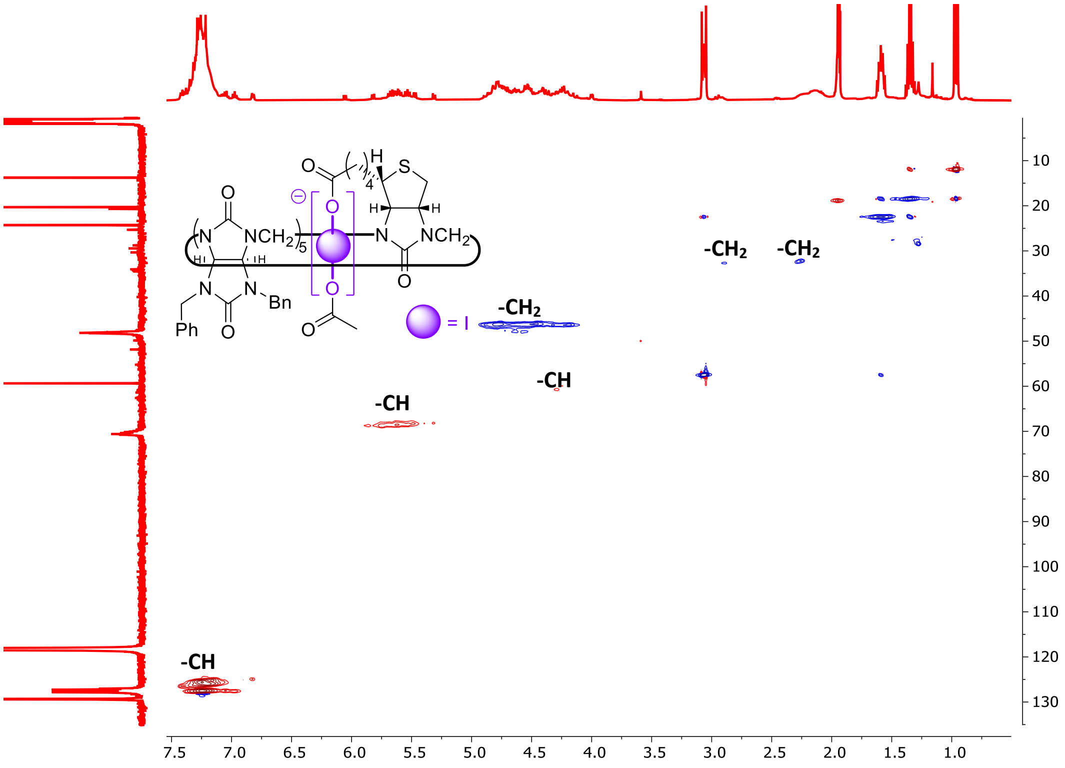

Figure S11. HSQC NMR spectrum (500 MHz, CD_3CN-d_3, 298.15 K) of **5**.

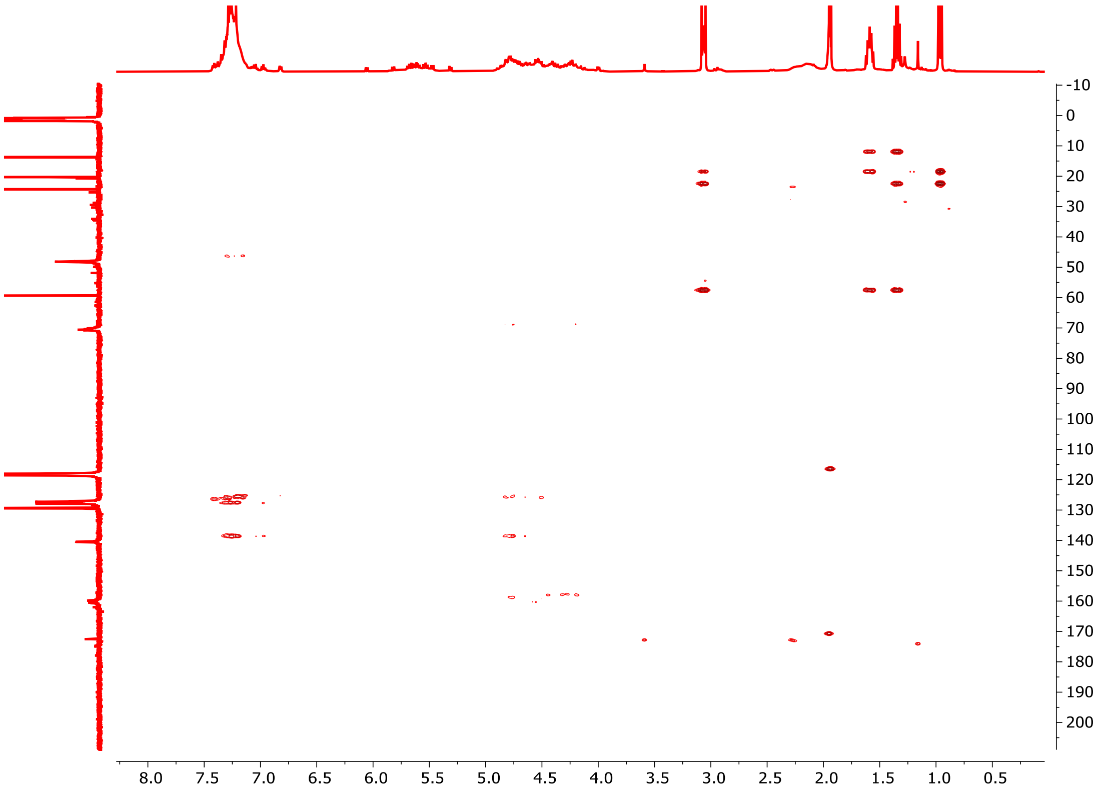

Figure S12. HMBC NMR spectrum (500 MHz, CD_3CN-d_3, 298.15 K) of **5**.

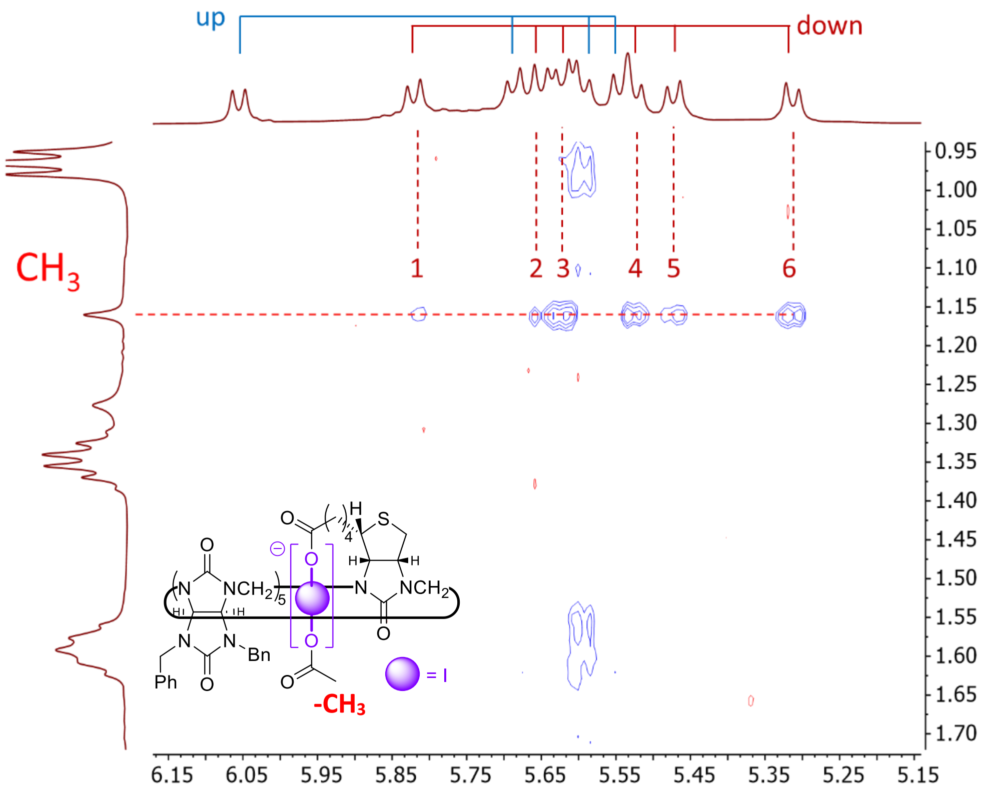

Figure S13. ROESY NMR spectrum (500 MHz, CD_3CN-d_3, 298.15 K) of **5**.

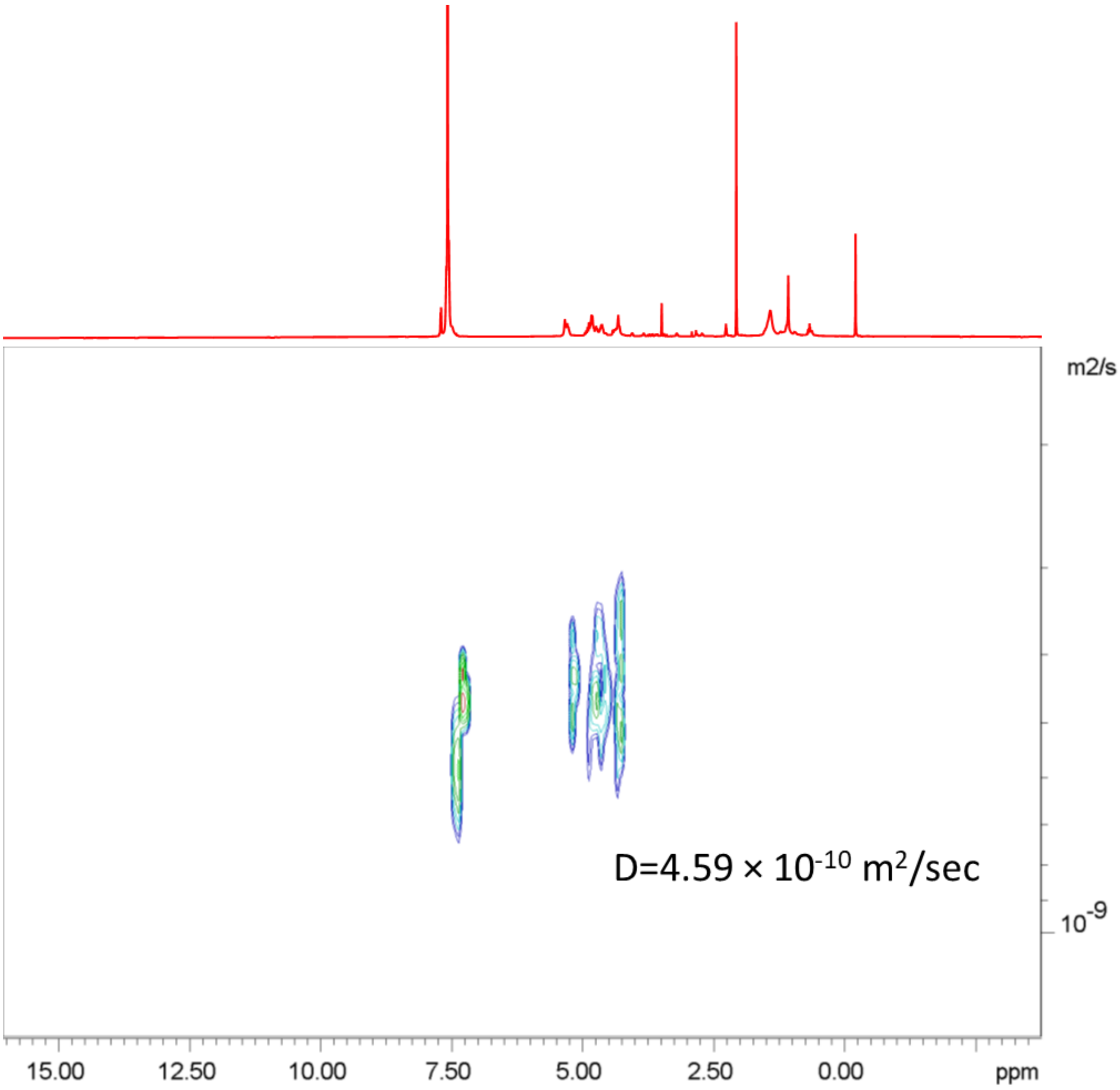

Figure S14. DOSY spectrum (500 MHz, $CDCl_3$-*d*, 298.15 K) of **1** (1mM).

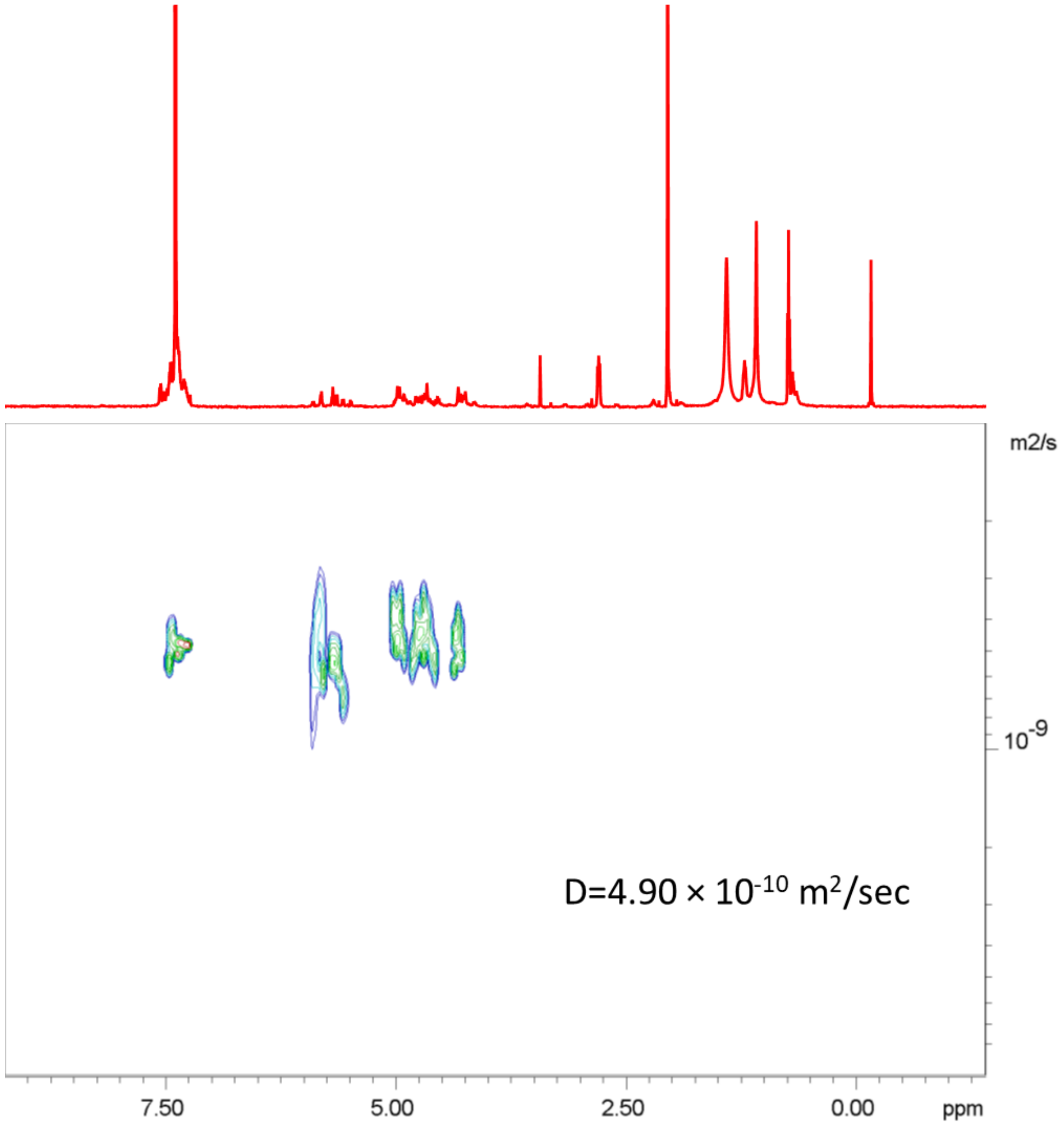

Figure S15. DOSY spectrum (500 MHz, $CDCl_3$-*d*, 298.15 K) of the $Cl^- \subset$**1** complex (1mM).

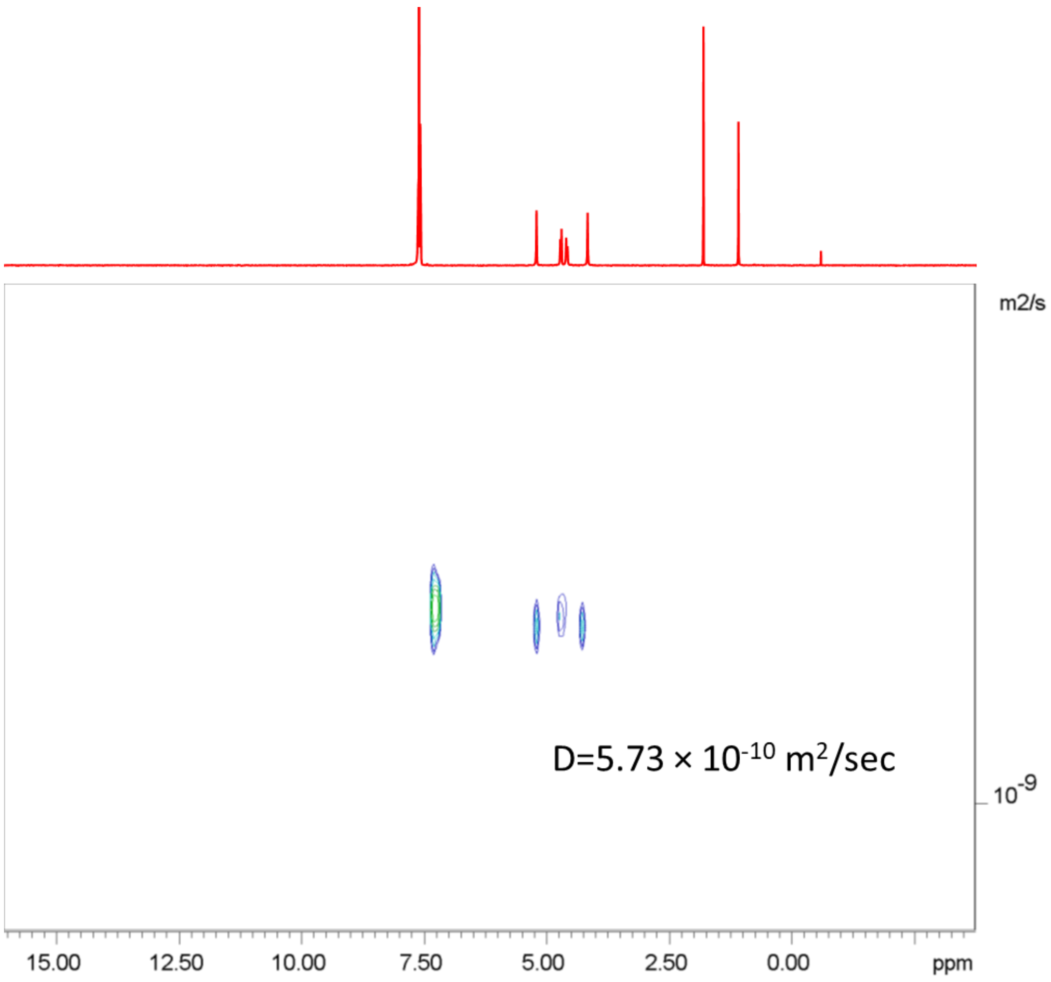

Figure S16. DOSY spectrum (500 MHz, $CDCl_3$-*d*, 298.15 K) of **BnBU** (1mM).

4. MALDI TOF spectra

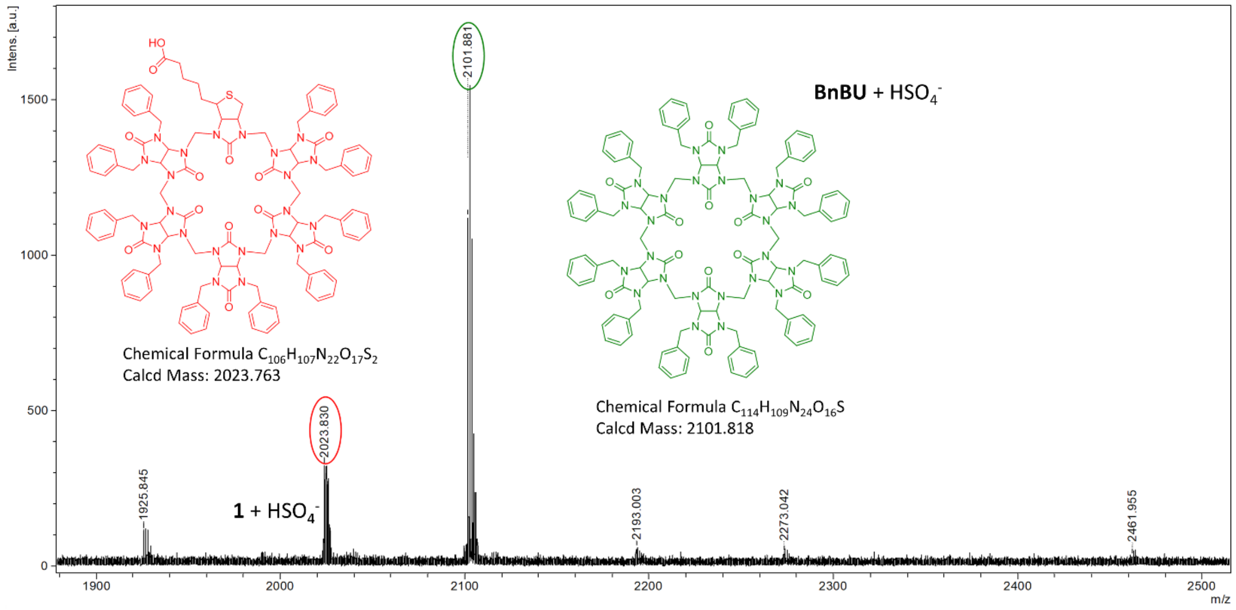

Figure S17. MALDI-MS spectrum (negative mode) of a crude reaction mixture for isolation of **1**

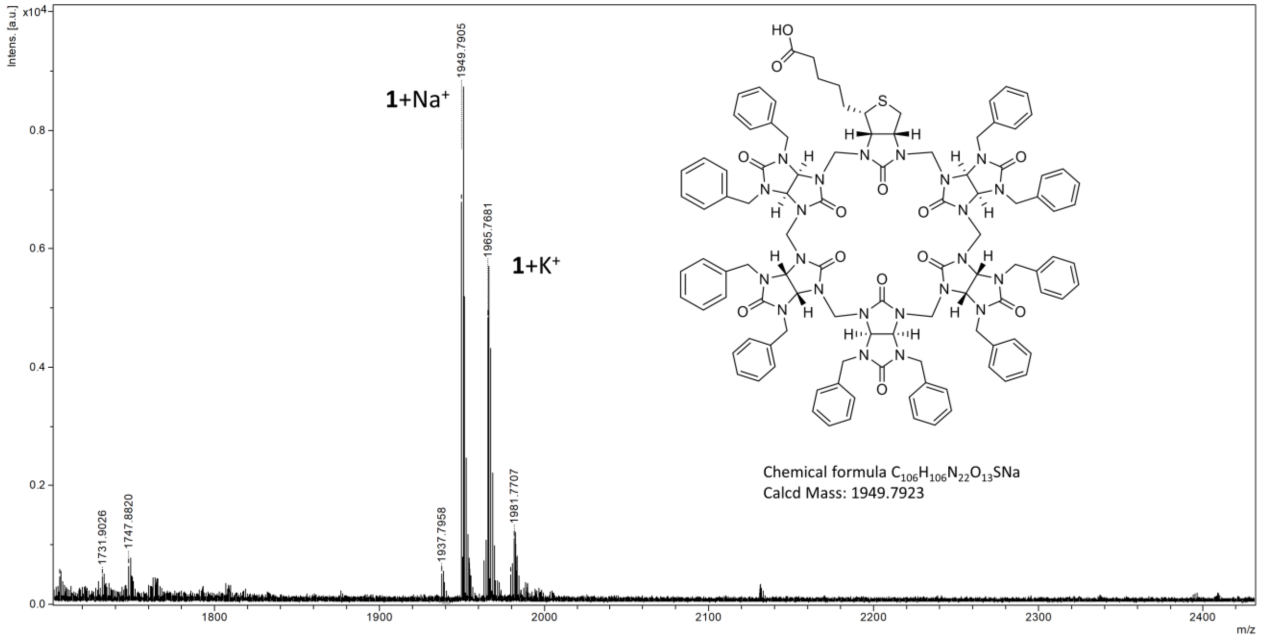

Figure S18. MALDI-MS spectrum (positive mode) of **1**.

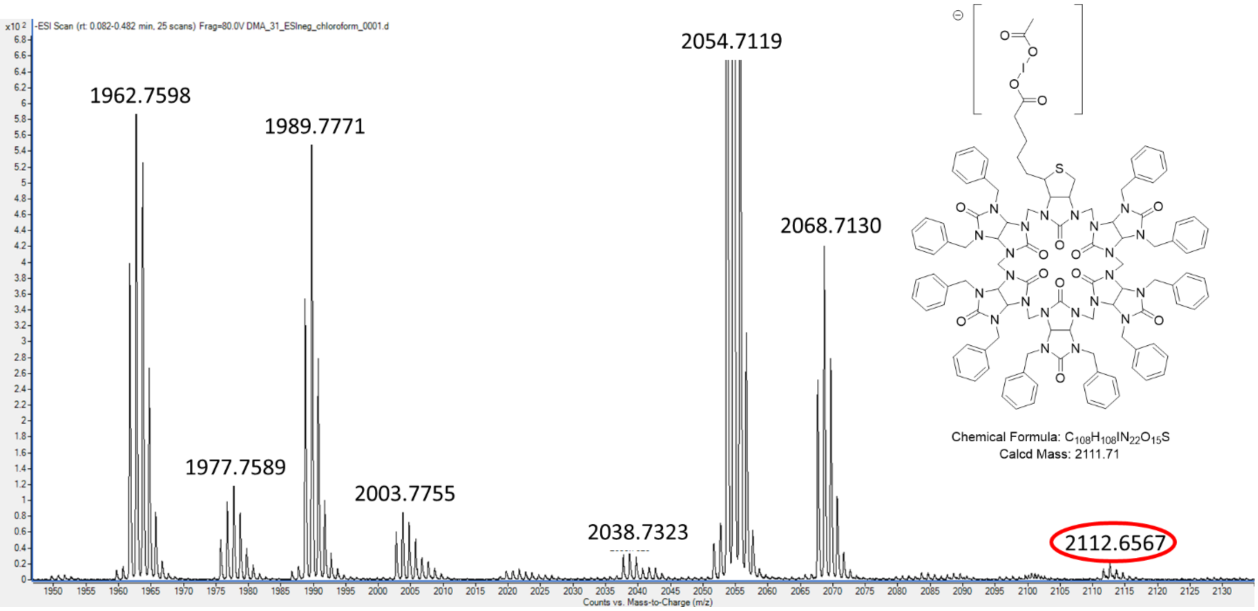

Figure S19. ESI Spectra (negative mode) of **5**.

5. Isothermal Titration Calorimetry (ITC)

An analysis using isothermal titration calorimetry (ITC) was conducted utilizing a MicroCal VP-ITC instrument from Malvern. The experiments were carried out at 298.15 K in a chloroform solvent. The heat responses recorded during the titration process are illustrated in the upper graph of each figure within this section. Each peak on the graph corresponds to the introduction of a 10 µL salt solution into the cell containing **1** alone or complexed with the competitor. In general, the lower graph depicts the cumulative heat released as a function of the total concentration of the ligand. The solid red line on the graph represents the best-fit line obtained through a least-squares analysis of the data. To examine the integrated heat effects, a single-site model was employed in nonlinear regression analysis. The association constant K_a and the standard binding enthalpy $\Delta H°$ were determined using experimental data matched to a theoretical titration curve. The standard free energy $\Delta G°$ and standard entropy $\Delta S°$ were obtained by means of the equation: $\Delta G° = \Delta H° - T\Delta S° = -RT \ln K_a$, where T is the absolute temperature and R is the molar gas constant (8.3145 J mol^{-1} K^{-1}).

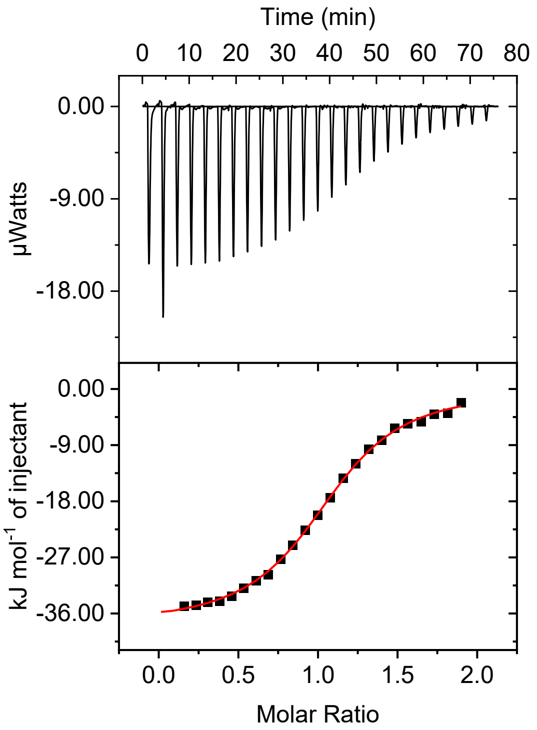

Figure S20. ITC titration of **1** (0.1 mM) with TBAMeSO3 (1.03 mM) in chloroform.

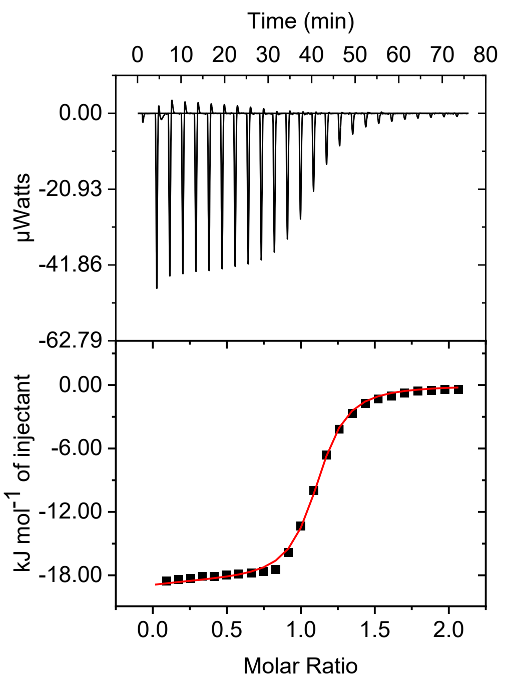

Figure S21. ITC titration of **1** (0.5 mM) with TBACl (5.04 mM) and TBAMeSO3 (1.03 mM) in chloroform.

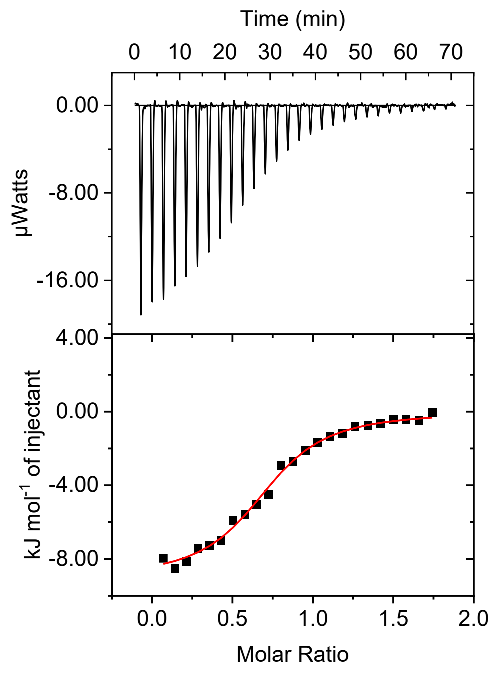

Figure S22. ITC titration of **1** (0.5 mM) with TBABr (5.04 mM) and TBACl (1 mM) in chloroform.

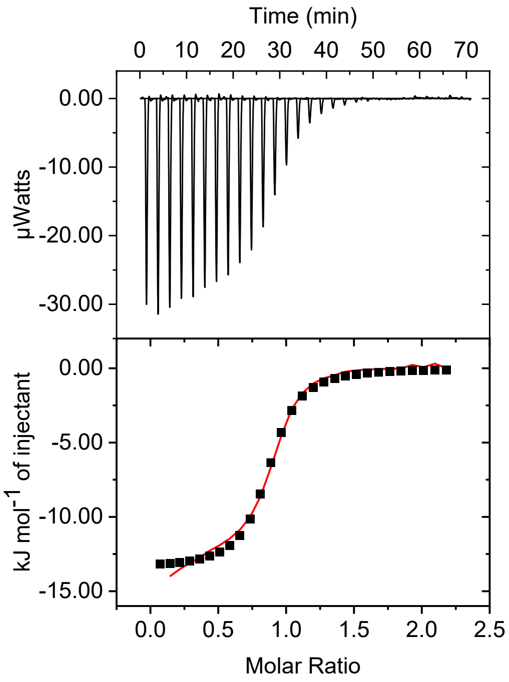

Figure S23. ITC titration **1** (0.5 mM) with TBAI (5.05 mM) and TBACl (1 mM) in chloroform.

6. Crystallography

Colorless crystals were prepared by slow diffusion of diethyl ether vapor into chloroform solution of **1** (1mM) and tetrabutylammonium chloride (1mM).

Diffraction data were collected on a Rigaku MicroMax-007 HF rotating anode CCD diffractometer using Mo K*α* radiation at 120 K. CrystalClear was used for data collection, CrysAlisPro for data reduction and absorption correction. The structures were solved by the direct methods procedure and refined by full matrix least-squares methods on F^2 using SHELXT and SHELXL. Crystal data and refinement parameters are gathered in Table S#. The supplementary crystallographic data for this paper can be obtained free of charge from The Cambridge Crystallographic Data Centre via www.ccdc.cam.ac.uk/data_request/cif.

Table S1. Crystallographic information for **1**.

**Crystal Data**	
**CCDC Deposition Number**	2266677
**Chemical formula**	C_{106} H_{106} Cl N_{22} O_{13} S
**Space Group**	P 2_1
**Temperature (K)**	120
**Cell Lengths**	**a** 16.8041(3) **b** 20.2203(3) **c** 39.8916(5)
**Cell Angles**	**a** 90 **b** 93.8333(14) **g** 90
**Cell Volume (Å)**	13524.2
Z	4
**R-Factor**	8.34
**Packing Coefficient**	0.589173

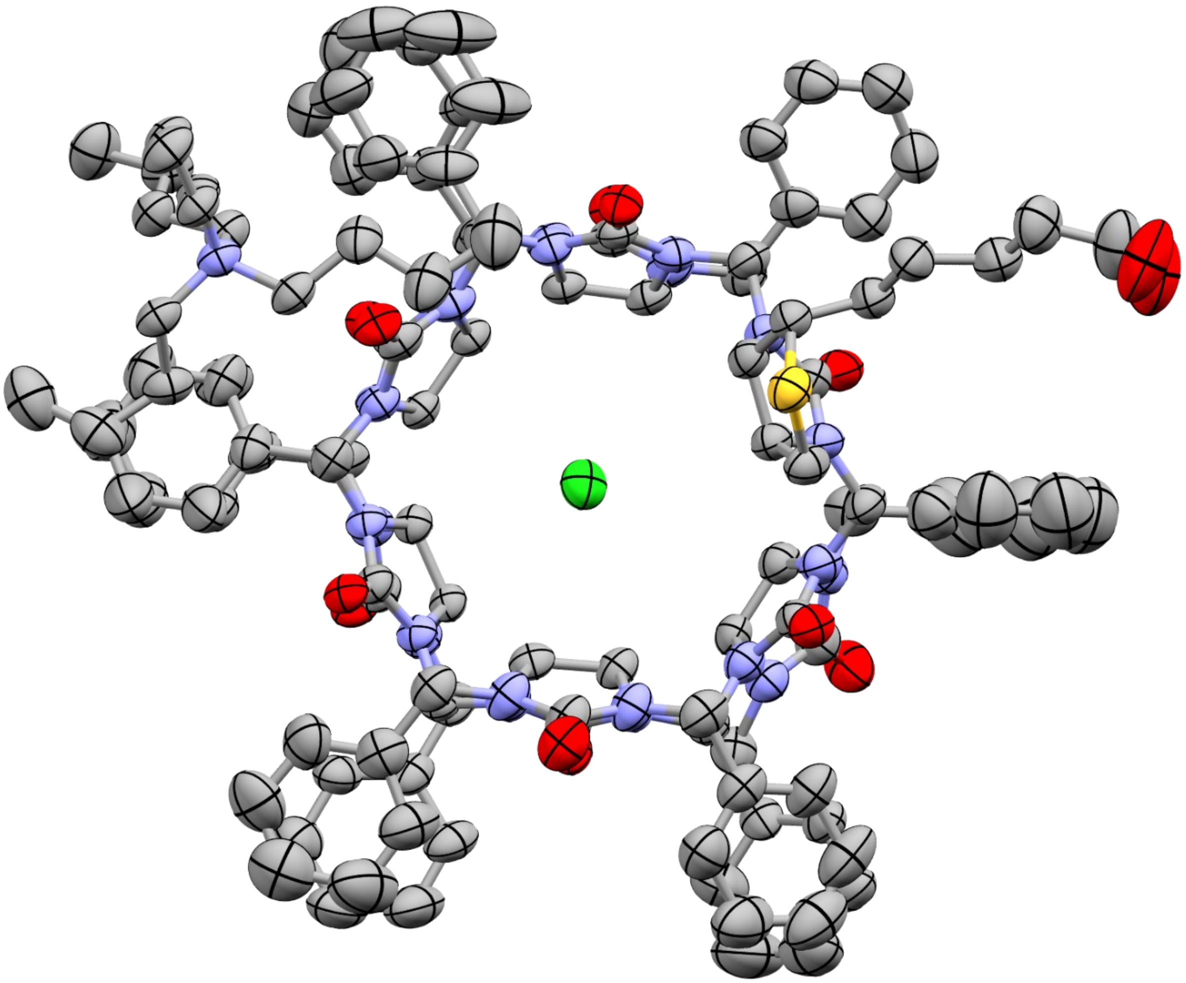

Figure S24. Molecular structure of the $Cl^{-}\subset$ **1** complex. Thermal ellipsoids are drawn at the 50% probability level.

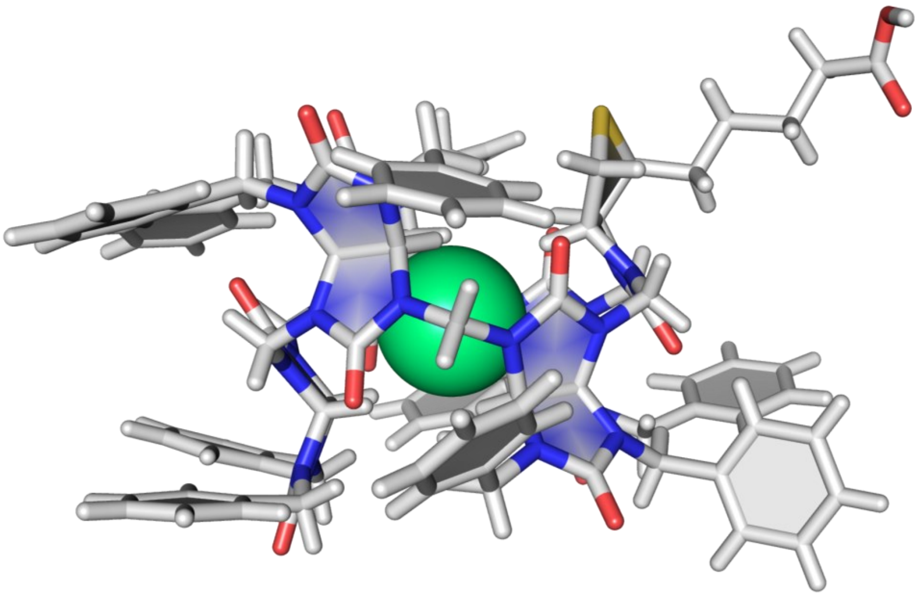

Figure S25. Side view of the Cl^{-}$\subset$ **1** complex.

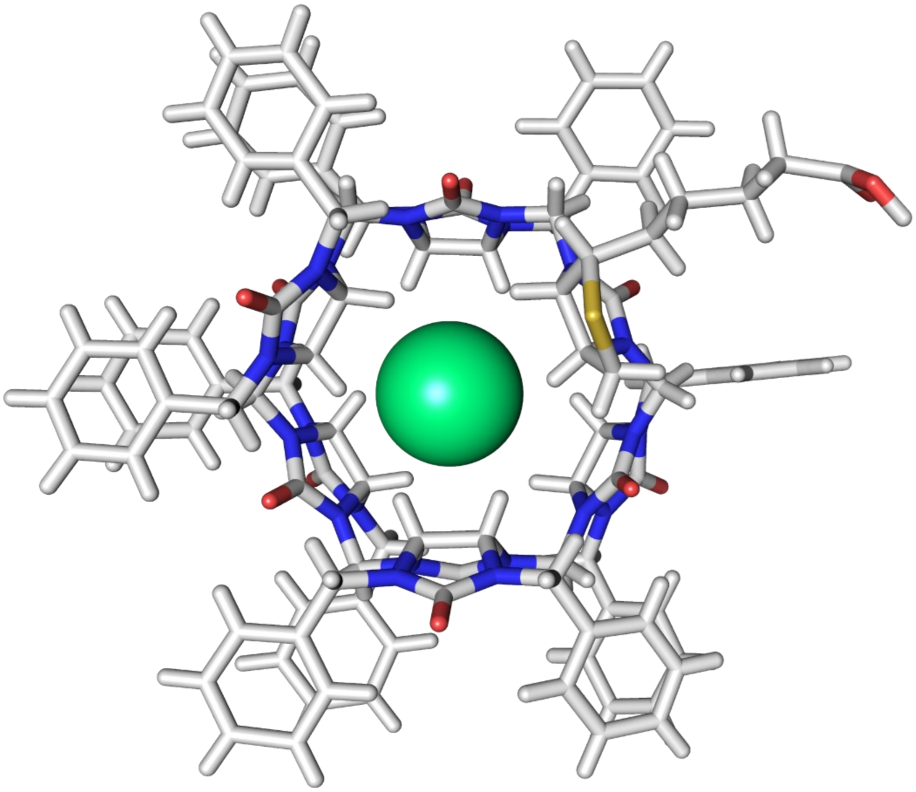

Figure S26. Top view of Cl^{-}$\subset$ **1** complex.

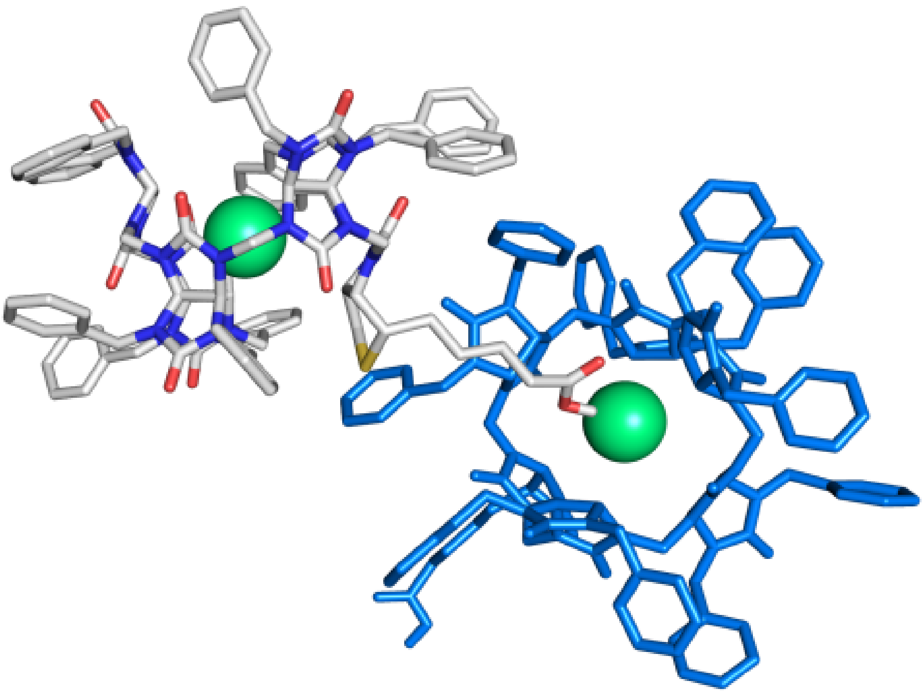

Figure S27. Dimer formed by Cl⁻⊂ **1** complex.

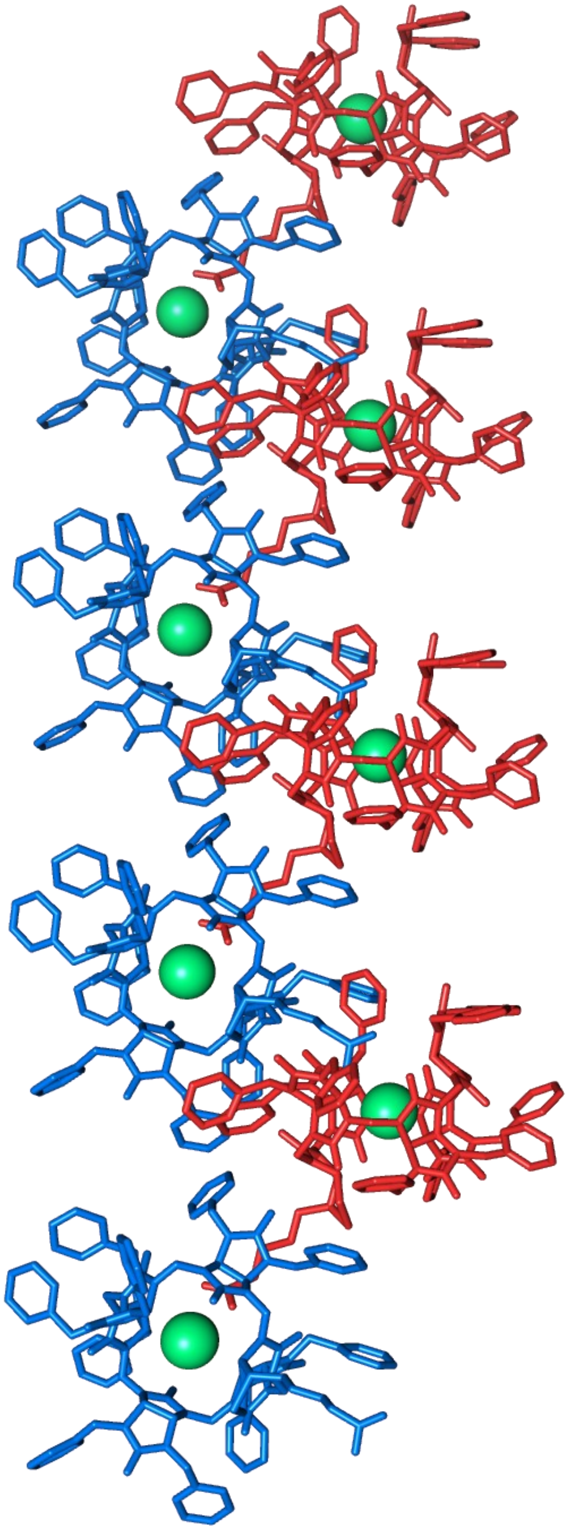

Figure S28. Supramolecular polymers formed by $Cl^{-}\subset$ **1** complex.

7. References

(1) Havel, V.; Svec, J.; Wimmerova, M.; Dusek, M.; Pojarova, M.; Sindelar, V. Bambus[n]Urils: A New Family of Macrocyclic Anion Receptors. *Org. Lett.* **2011**, *13* (15), 4000–4003. https://doi.org/10.1021/ol201515c.

(2) Muñiz, K.; García, B.; Martínez, C.; Piccinelli, A. Dioxoiodane Compounds as Versatile Sources for Iodine(I) Chemistry. *Chemistry – A European Journal* **2017**, *23* (7), 1539–1545. https://doi.org/10.1002/chem.201603801.

(3) Mauro, A. D.; Kokan, Z.; Šindelář, V. Dynamic [1]Rotaxanes via a Reversible Covalent Bond and Host–Guest Anion Recognition. *Chem. Commun.* **2022**, *58* (23), 3815–3818. https://doi.org/10.1039/D2CC00779G.
